# Combinations of Abiotic Factors Differentially Alter Production of Plant Secondary Metabolites in Five Woody Plant Species in the Boreal-Temperate Transition Zone

**DOI:** 10.3389/fpls.2018.01257

**Published:** 2018-09-05

**Authors:** John L. Berini, Stephen A. Brockman, Adrian D. Hegeman, Peter B. Reich, Ranjan Muthukrishnan, Rebecca A. Montgomery, James D. Forester

**Affiliations:** ^1^Conservation Biology Graduate Program, University of Minnesota, St. Paul, MN, United States; ^2^Institute on the Environment, University of Minnesota, St. Paul, MN, United States; ^3^Department of Horticultural Science, The Microbial and Plant Genomics Institute, University of Minnesota, St. Paul, MN, United States; ^4^Department of Forest Resources, University of Minnesota, St. Paul, MN, United States; ^5^Hawkesbury Institute for the Environment, Western Sydney University, Penrith, NSW, Australia; ^6^Department of Fisheries, Wildlife, and Conservation Biology, University of Minnesota, St. Paul, MN, United States

**Keywords:** phytochemical turnover, PSM diversity, untargeted metabolomics, balsam fir, beaked hazel, paper birch, red maple, trembling aspen

Plant secondary metabolites (PSMs) are a key mechanism by which plants defend themselves against potential threats, and changes in the abiotic environment can alter the diversity and abundance of PSMs. While the number of studies investigating the effects of abiotic factors on PSM production is growing, we currently have a limited understanding of how combinations of factors may influence PSM production. The objective of this study was to determine how warming influences PSM production and how the addition of other factors may modulate this effect. We used untargeted metabolomics to evaluate how PSM production in five different woody plant species in northern Minnesota, United States are influenced by varying combinations of temperature, moisture, and light in both experimental and natural conditions. We also analyzed changes to the abundances of two compounds from two different species – two resin acids in *Abies balsamea* and catechin and a terpene acid in *Betula papyrifera*. We used permutational MANOVA to compare PSM profiles and phytochemical turnover across treatments and non-metric multidimensional scaling to visualize treatment-specific changes in PSM profiles. We used linear mixed-effects models to examine changes in phytochemical richness and changes in the abundances of our example compounds. Under closed-canopy, experimental warming led to distinct PSM profiles and induced phytochemical turnover in *B. papyrifera*. In open-canopy sites, warming had no influence on PSM production. In samples collected across northeastern Minnesota, regional temperature differences had no influence on PSM profiles or phytochemical richness but did induce phytochemical turnover in *B. papyrifera* and *Populus tremuloides*. However, warmer temperatures combined with open canopy resulted in distinct PSM profiles for all species and induced phytochemical turnover in all but *Corylus cornuta*. Although neither example compound in *A. balsamea* was influenced by any of the abiotic conditions, both compounds in *B. papyrifera* exhibited significant changes in response to warming and canopy. Our results demonstrate that the metabolic response of woody plants to combinations of abiotic factors cannot be extrapolated from that of a single factor and will differ by species. This heterogeneous phytochemical response directly affects interactions between plants and other organisms and may yield unexpected results as plant communities adapt to novel environmental conditions.

## Introduction

Plant secondary metabolites (PSMs) are one of the primary ways in which plants respond to environmental variability, and regulation of PSM production is strongly influenced by the local environment (Wink, 1988; Bennett and Wallsgrove, 1994; Bray et al., 2000; Hirt and Shinozaki, 2003). Many interactions between plants and other organisms are mediated by PSMs (Farmer, 2001; Karban et al., 2006; Karban, 2008), and thus, the biochemical mechanisms that influence these interactions are modulated, at least in part, by the presence, absence, or magnitude of various environmental factors (DeLucia et al., 2012; Jamieson et al., 2012). For instance, changes in the amount and seasonality of precipitation have been shown to influence concentrations of cyanogenic glycosides (Gleadow and Woodrow, 2002; Vandegeer et al., 2013), and elevated concentrations of atmospheric CO_2_ often result in increased concentrations of condensed tannins (Lindroth, 2012). Evidence is mounting that recent warming may also influence the production of PSMs (Kuokkanen et al., 2001).

Studies investigating the influence of warming on PSM production suggest that temperature-induced changes to PSMs may be species, compound, or even context dependent. For example, warming has been shown to have no effect on levels of phenolics in red maple (*Acer rubrum*, Williams et al., 2003), Norway spruce (*Picea abies*, Sallas et al., 2003), and Scots pine (*Pinus sylvestris*, Sallas et al., 2003) but resulted in decreased levels of phenolics in dark-leaved willow (*Salix myrsinifolia*, Veteli et al., 2006) and silver birch (*Betula pendula*, Kuokkanen et al., 2001). Additionally, warming has been shown to increase levels of terpene-based compounds in Norway spruce (Sallas et al., 2003), Ponderosa pine (*Pinus ponderosa*, Constable et al., 1999), and Scots pine (Sallas et al., 2003) but has been shown to both increase (Constable et al., 1999) and decrease (Snow et al., 2003) levels of monoterpenes in Douglas fir (*Pinus menziesii*). While evidence of warming-induced changes to phytochemistry is important to our understanding of how plants will respond to future climates, in natural settings, elevated temperature often combines with other abiotic conditions to influence PSM production and potentially modulate any changes to phytochemistry that might otherwise be induced by warming alone.

As temperatures continue to rise, global precipitation patterns are expected to shift (Hurrell, 1995; Alexander et al., 2006; IPCC, 2014) and light availability to understory plants will likely be altered due to changes in the frequency and intensity of forest disturbance patterns (Canham et al., 1990; Dale et al., 2001). While variability in each of these environmental factors has been shown to influence production of PSMs on their own (Bryant et al., 1983; Dudt and Shure, 1994; Pavarini et al., 2012), combinations of factors can have a distinct effect (Rizhsky et al., 2002, 2004; Mittler, 2006; Zandalinas et al., 2018). Moreover, plant responses to combinations of abiotic factors can be either synergistic or antagonistic (Bonham-Smith et al., 1987; Mittler, 2006; Zandalinas et al., 2018). For example, drought has been shown to enhance cold tolerance (Cloutier and Andrews, 1984), but also exacerbate a plant’s intolerance of high temperatures (Rizhsky et al., 2002). Further, different combinations of salinity and high temperatures have been shown to have both positive and negative influences on the metabolism of reactive oxygen species and stomatal response (Zandalinas et al., 2018). Regardless, the vast majority of current research remains focused on the influences of individual conditions rather than considering potential interactions among them.

Until recently, the majority of studies investigating the potential influence of different abiotic factors largely considered the effects of these factors on individual compounds or small groups of compounds. However, individual metabolites rarely, if ever, function in isolation (Gershenzon et al., 2012). Rather, the influence of any one compound is dependent on conditions within the local environment, as well as the relative abundance of numerous other metabolites within a plant’s array of chemical constituents (Dyer et al., 2003; Richards et al., 2010; Gershenzon et al., 2012; Jamieson et al., 2015). Thus, understanding how changes in the abiotic environment will influence a plant’s metabolic profile is important for interpreting how these changes will influence the abundance and biological role of individual compounds as well.

Phytochemical diversity influences how effective plants are when defending against a range of threats (Gershenzon et al., 2012; Frye et al., 2013; Richards et al., 2015). Compounds may act synergistically, thereby forming mixtures that can provide enhanced protection against potential hazards (Gershenzon, 1984; Harborne, 1987; Gershenzon et al., 2012). Indeed, recent evidence suggests that the number of individual compounds comprising a plant’s phytochemical profile can even influence local biological diversity via the influence of changes in toxicity on rates of herbivory (Richards et al., 2015). Increased diversity of secondary metabolites may also allow for more precise communication between plants, thereby allowing for more robust protection against a range of conditions (Iason et al., 2005; Poelman et al., 2008; Gershenzon et al., 2012; Frye et al., 2013). Two metrics that are useful for assessing changes in phytochemical diversity are “phytochemical richness” (i.e., the absolute number of compounds produced) and “phytochemical turnover” (i.e., the degree of overlap among the compounds produced), as both measures provide different insights into the metabolic response of plants to a range of abiotic conditions.

Variability in phytochemistry, even within the same species, may influence ecosystem structure and function through an array of chemically driven ecological effects (Bukovinszky et al., 2008; Gillespie et al., 2012; Sedio et al., 2017). The growth-differentiation balance hypothesis (GDBH) suggests that as the local environment becomes increasingly stressful, growth processes will become limited and the production of PSMs will increase until the point that PSM production also becomes limited by resource acquisition/availability (Lerdau et al., 1994). While phytochemical diversity has not been explicitly tested in light of the GDBH, studies have shown that herbivore-induced secondary chemistry can be completely suppressed in some woody species under a range of abiotic conditions (Lewinsohn et al., 1993), rendering them vulnerable to further invasion by pests and pathogens. While the number of studies investigating the effects of warming and other abiotic conditions on PSM production is rapidly growing, we currently have a limited understanding of how different abiotic factors may interact to influence phytochemical diversity (Bidart-Bouzat and Imeh-Nathaniel, 2008; Jamieson et al., 2012, 2015). The objective of this study was to determine how elevated temperatures may influence the production of PSMs and to evaluate how the addition of other abiotic factors may modulate this effect.

While a targeted approach uses standard model compounds to identify and observe changes in specific compounds selected a priori, an untargeted (i.e., global) approach makes no assumptions regarding specific metabolites, and therefore, allows one to observe global changes across the entire metabolic profile. Thus, the strength of an untargeted approach lies in the potential to discover unanticipated changes in metabolic profiles as a result of environmental perturbations (Crews et al., 2009). Although untargeted metabolomics have been used in medicine for clinical diagnosis of various diseases, including numerous forms of cancer (Sreekumar et al., 2009; Jain et al., 2015), this study is among the first to apply this method to an ecological setting.

We used an untargeted metabolomics approach to evaluate how the phytochemical profiles of five different woody plant species are influenced by temperature, soil moisture, and light. Specifically, we tested the hypothesis that elevated temperatures alter the production of PSMs by leading to phytochemical profiles that are distinct from those found at ambient temperature (H1) and that warming will change phytochemical diversity via reductions in phytochemical richness or a high degree of turnover (H2). We also tested the hypothesis that the addition of other abiotic factors, specifically high light and drought, will either magnify or nullify temperature-induced changes in phytochemical profiles and PSM diversity (H3). Finally, because individual compounds may vary greatly in response to heterogeneity in the abiotic environment, we identified two ‘example compounds’ from balsam fir (*Abies balsamea –* two unspecified di-terpene resin acids) and paper birch (*Betula papyrifera* – catechin and another unspecified di-terpene resin acid) and analyzed the effects of different sets of abiotic factors (high-temperature, light, and drought) on their relative abundance. Specifically, we tested the hypothesis that individual compounds will respond to different conditions and combinations of conditions by either increasing or decreasing in relative abundance, potentially in a non-uniform and unpredictable manner (H4).

## Materials and Methods

### Experimental Design

The Boreal Forest Warming at an Ecotone in Danger (B4WarmED) project is an ecosystem experiment that simulates both above- and below-ground warming in a boreal forest community. The experiment was conducted at Cloquet Forestry Center (CFC; Cloquet, MN, United States) and was initiated in 2008. The experimental design consists of a 2 (overstory – open and closed) × 3 (warming – ambient, ambient +1.7°C, and ambient +3.4°C) × 2 (precipitation – ambient and ambient -40%) factorial design with six replicates (two per block) per treatment combination, for a total of 72 – 7.1 m^2^ plots (Rich et al., 2015). Within each plot, 121 seedlings of 11 tree species were planted into the remaining herbaceous vegetation in a gridded design (Rich et al., 2015). Above-ground biomass was warmed using a Temperature Free-Air-Controlled Enhancement System (T-FACE) and below-ground biomass was warmed via buried resistance-type heating cables (Rich et al., 2015). Above- and below-ground temperatures have been monitored and logged at 15-min intervals since spring 2008. In 2012, event-based rain exclosures were installed on nine plots in the open overstory replicates of the warming experiment, which allowed for safe and reliable removal of rainfall. Mean annual rainfall exclusion from June to September ranges from 42 to 45%.

We collected plant samples from the B4WarmED project during two different time periods. On July 14, 2013, we collected samples of balsam fir and paper birch that were grown under closed overstory and three warming treatments, and on July 15, 2014, we collected samples of balsam fir, paper birch, trembling aspen (*Populus tremuloides*), and red maple (*Acer rubrum*) grown under open overstory in the three warming treatments and two precipitation treatments. Where possible, we collected recent-growth foliar tissue from two plants per species within each replicate plot. However, some replicates contained either one or no plants with enough leaf tissue to sample. Samples sizes were particularly small during 2014, so we were forced to group individual warming treatments (ambient, +1.7°C, +3.4°C) into a binary response (ambient temperature vs. elevated temperature). All plant samples were collected within a 2-h time period. Upon collection, samples were flash frozen with dry ice, and subsequently stored in a -80°C freezer to minimize chemical degradation. We broadly refer to samples collected from the B4WArmED project as our “experimental” samples.

**FIGURE 1:**
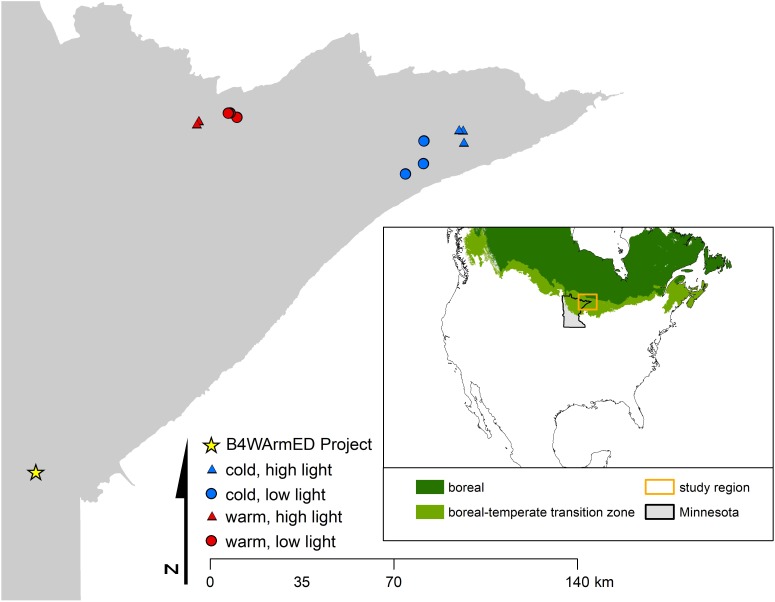
Location of observational sites and the B4WarmED Project at the University of Minnesota’s Cloquet Forestry Center. The number of replicate plots for each set of abiotic conditions is *n* = 3, and where only two can be seen for a given combination of abiotic factors (i.e., temperature + light conditions), locations are close enough in proximity that they appear to overlap when viewed at a broad scale. Inset map identifies the approximate location of the study area within the state of Minnesota and the boreal-temperate transition zone (Brandt, 2009).

To investigate how temperature and light conditions may interact to influence phytochemical production in a natural forest environment, we collected samples of balsam fir, paper birch, trembling aspen, and beaked hazel (*Corylus cornuta*) from open and closed canopy environments across two regions in northeastern Minnesota (**Figure 1**). These regions exhibit differences in mean-maximum summer temperature (maximum daily temperature averaged across June, July, and August) of approximately 5.5°C (**Supplementary Table S1**). On July 14, 2015, we collected a minimum of 3 biological replicates from each species within each set of abiotic conditions. The sampling design consists of a 2 (overstory – open and closed) × 2 (temperature – warm and cool) design with three plot replicates per treatment combination, for a total of 12 – 400 m^2^ plots. Open-canopy plots allowed us to evaluate high-light conditions on production of PSMs and were located in areas that were clear-cut in 2006 (i.e., open overstory), while closed-canopy plots were located in areas that experienced no known overstory disturbance since at least 1985 (i.e., closed overstory). Thus, light conditions for all plots were based on whether the overstory was open (i.e., high light) or closed (i.e., low light). Temperature logger data collected for a parallel study from similar plot types suggest that average high temperatures from May 1, 2015 to July 14, 2015 ranged from 30.4°C in low-light plots in the cool region to 36.6°C in high-light plots in the warm region. All field samples were collected on the same day, within an 8-h period. Upon collection, samples were flash frozen with dry ice, and subsequently stored in a -80°C freezer. For brevity, we occasionally refer to samples collected throughout northeast Minnesota as “observational” samples.

### Study Organisms

Balsam fir is a mid- to large-sized species of conifer, growing to 26 m in height, with shallow roots (Smith, 2008). It is highly vulnerable to drought, fire, and spruce budworm (*Choristoneuro fumiferana*) infestations (Engelmark, 1999), and modest climate warming has been shown to decrease net photosynthesis and growth by as much as 25% (Reich et al., 2015). Paper birch can grow to 28 m in height (Smith, 2008) and is drought and shade intolerant (Iverson and Prasad, 1998; Iverson et al., 2008). While it can grow rapidly and live to be 250 years of age, seedlings need significant light to prosper (Kneeshaw et al., 2006). Elevated temperatures have been shown to influence foliar nitrogen, lignin, and condensed tannins in both paper birch and trembling aspen with the specific response varying as a function of species and climate (Jamieson et al., 2015). Trembling aspen is one of the most widespread tree species in North America and occurs on a wide-range of soil types and in various climatic conditions (Smith, 2008). It is sensitive to both drought and shade (Iverson and Prasad, 1998; Iverson et al., 2008) and may become increasingly vulnerable to other potential stressors under conditions of drought and high temperatures (Worrall et al., 2008). Red maple is a moderately large tree, growing to 29 m in height and is known to be tolerant to a wide-range of precipitation conditions, from drought to seasonal flooding (Smith, 2008). While this species is expected to prosper under future climate scenarios (Iverson and Prasad, 1998; Iverson et al., 2008) and performed well under experimental warming (Reich et al., 2015), both prolonged flooding and severe drought have been shown to result in senescence and decreased growth, respectively (Nash and Graves, 1993). Beaked hazel, a shade-tolerant shrub that can grow to 4 m tall, is a common understory species in both conifer and deciduous forests and occurs almost exclusively in fire prone habitats (Smith, 2008). Beaked hazel is highly sensitive to fire and previous work suggests that growth may be limited by soil moisture (Johnston and Woodard, 1985).

### Metabolite Analysis

Tissue samples were lyophilized for 72 h and then homogenized and extracted using 25 mg (+/-2.5 mg) of each sample. Homogenization and extraction were performed for 5 min at a frequency of 1500 Hz with 1 ml of 70% isopropyl alcohol at –20°C using a bead mill and 2.8 mm tungsten carbide beads (Sped Sample Prep GenoGrinder 2010, Metuchen, NJ, United States). Samples were then subjected to centrifugation at 16,000 ×*g* for 5 min. The supernatant was then removed and subjected to an additional centrifugation step, 16,000 ×*g* for an additional 5 min, and the supernatant was collected for subsequent analysis. Finally, 20 μL of each supernatant sample was removed and pooled to use as a control. All samples were then stored at –80°C.

We analyzed samples with liquid chromatography mass spectrometry (LC-MS) using an Ultimate 3000 UHPLC (ultra-high-performance liquid chromatography) system coupled to a Q Enactive hybrid quadrupole-Orbitrap mass spectrometer with a heated electrospray ionization (HESI) source (Thermo Fisher Scientific, Bremen, Germany). We injected 1 μL of each sample per analysis onto an ACQUITY UPLC HSS T3 column, 100 Å, 1.8 μm, 2.1 mm × 100 mm (Waters, Milford, MA, United States) using a gradient composed of solvents A: 0.1% formic acid and B: acetonitrile. Specifically, 0–2 min, 2% B; 6 min, 24% B; 9 min, 33% B; 12 min, 65% B; 16 min, 80% B; 20 min 93% B; 21 min 98% B; 22 min 98% B; 23 min 2% B; 23–25 min 2% B. Samples were analyzed in a randomized order to minimize systematic bias from instrument variability and carryover. Full-scan analysis was performed using positive/negative ion polarity switching, a 115–1500 *m/z* scan range, a resolution of 70,000 (at *m*/*z* 200), maximum fill times of 100 ms, and target automatic gain control (AGC) of 1 × 10^6^ charges. Ion fragmentation was performed using a higher-energy collision dissociation (HCD) cell and resulting MS/MS data were collected using a resolution of 17,500, maximum fill times of 100 ms, and an AGC target of 2 × 10^5^ charges. Normalized collision energies (NCE) ranged from 10 to 45 in increments of 5. All data were collected using Xcalibur version 2.2 (Thermo Fisher Scientific, Bremen, Germany).

### Example Compounds

To determine which chemical features varied consistently and significantly among each treatment and species group, we aligned, smoothed, background subtracted, and analyzed all chromatographic data using analysis of variance (α = 0.001) via Genedata 7.1 (Genedata, Basel, Switzerland). We assigned putative metabolite identities only to the features found to be significantly abundant (ANOVA, α = 0.001) with an exact mass and higher-energy collisional dissociation (HCD) MS/MS fragmentation spectra. We determined molecular formulae by using exact mass to calculate the most probable elemental composition for each feature (**Supplementary Table S2**). We then manually interpreted HCD spectra collected at numerous collision energies (**Supplementary Figures S1–S3**), and compared these to the MassBank database using MetFusion (Gerlich and Neumann, 2013). Where possible, we confirmed the identity of individual compounds via comparison to an authenticated standard (Sigma-Aldrich) and assigned other putative identities by matching molecular formulae to those of previously observed metabolites in *Betula* (Julkunen-Tiitto et al., 1996) and *Abies* (Otto and Wilde, 2001). Specifically, we analyzed changes in the relative abundance of catechin and an unspecified terpene acid in paper birch and two unspecified diterpene resin acids in balsam fir. The identification of catechin was confirmed by comparison of accurate mass, LC-retention and MS/MS fragmentation properties of commercially available standard compounds for both catechin and its frequently associated isomer epicatechin which were distinguishable by chromatographic separation. There has been a great deal of work investigating the biological and ecological activity of catechin and terpenoid-based metabolites (Tahvanainen et al., 1985; Gershenzon and Croteau, 1992; Berg, 2003; Stolter et al., 2005); and as a result, we expect our results regarding these compounds to be broadly relevant.

### Data Processing and Statistical Analysis

Data processing and statistical analyses were conducted using R 3.5.0 (R Core Team, 2017). To initiate data processing, we used the *xcmsRaw* function in the *xcms* package (Smith et al., 2006; Tautenhahn et al., 2008; Benton et al., 2010) to read our raw mzML files into R. After separating our data by polarity using the *split* function in the *base* package, we used the *findPeaks.centwave* function for peak detection, which we parameterized as follows: *ppm* = 2, *peakwidth* = c(5,20), *prefilter* = c(1,15000000), *mzCenterFun* = “apex,” *integrate* = 1, *mzdiff* = -0.001, *fitgauss* = F, *snthresh* = 10. Once peak detection was complete, we trimmed the resulting polarity-specific data frames based on retention time and retained only those peaks detected between 1 and 21 min.

A major shortfall of employing LC-MS to perform “untargeted profile analysis,” as we did here, is the production of two independent but partially overlapping datasets resulting from ion polarity switching. While polarity switching is useful for detection of features that can only be detected via either positive or negative ionization, some features are detectable under both ionization modes, therefore resulting in two independent data sets containing a small subset of common features. Moreover, interpretation of statistical results is challenging due to the presence of parallel sets of analyses with common features contributing to both. To alleviate these issues, we combined positive and negative polarities using the *find.matches* function in the *Hmisc* package (Harrell and Dupont, 2018). The *find.matches* function allows one to identify which rows in a data matrix align with those in a separate, identically formatted matrix by allowing the user to define a tolerance level for the numerical columns in each matrix. Thus, to determine our common features in the positive and negative ionization datasets that result from LC-MS, we created two matrices for positive and negative polarity, containing three separate columns – the mass of each detected peak, an assigned name for each peak, and retention time. To ensure that corresponding features from each ionization mode were capable of alignment, we subtracted 2.1046, roughly the mass of two protons, from all masses in the positive polarity dataset. For those features identified as common among both ionization modes, we retained peak data from the polarity exhibiting greatest mean intensity across all samples. We then assigned new peak names to identify which peaks were present in either positive or negative polarity vs. those that were found in both. All output created using the *find.matches* function was manually checked to ensure that all peaks identified as having a match in one polarity, had their mate identified as a match in the other.

We used permutational MANOVA (perMANOVA, Anderson, 2001) to compare PSM profiles between abiotic conditions. When analyzing PSM profiles, differences were estimated using Canberra dissimilarity matrices (Dixon et al., 2009). Analysis was performed with the *adonis* function (from the vegan package, Oksanen et al., 2015), which allowed us to account for our blocked sampling design via the *strata* argument. Both differences in the centroids among conditions or differences in multivariate dispersion can lead to statistically significant results when using perMANOVA. To determine whether differences among centroids were contributing to perMANOVA results, we created mean dissimilarity matrices using the *meandist* function and we used the *betadisper* function to assess multivariate homogeneity of variance (i.e., dispersion, Oksanen et al., 2015). We used non-metric multidimensional scaling (NMDS, Kruskal, 1964) to visualize differences in PSM profiles among conditions, which we performed using the *metaMDS* function in the vegan package (Oksanen et al., 2015). We set our dimensionality parameter (k) to 2 and projected condition-specific effects onto NMDS plots using the *ordiellipse* function to plot 95% confidence ellipses based on standard error (Oksanen et al., 2015).

To evaluate treatment-induced changes to PSM diversity, we calculated phytochemical richness based on the presence and absence of individual compounds, then tested the main effect of treatment on richness with block (experimental samples) or site ID (observational samples) as our random effect using linear mixed-effects models (*lme* function within the *nlme* package, Pinheiro et al., 2015). To analyze phytochemical turnover (i.e., the degree of overlap between the phytochemical profiles of individual plants across and between conditions), we created dissimilarity matrices based on binary datasets representing the presence or absence of each feature using Jaccard’s Index. We evaluated condition-specific differences in phytochemical turnover using perMANOVA via the *adonis* function, and evaluated the influence of multivariate centroids and homogeneity of variance on perMANOVA results as detailed above (Anderson, 2001; Oksanen et al., 2015).

Weather data from CFC shows that ambient air temperature, cumulative precipitation from 1 January to collection date, and leaf surface temperature were not statistically different between 2012 and 2013 or between specific sample sets (2013 – closed overstory, 2014 – open overstory). However, soil moisture and soil temperature vary strongly between years and sample sets, and differences between experimental and observational samples are likely to be even greater. Thus, samples collected during different years were analyzed independently of one another as individual data sets.

For analytical and visualization purposes, the condition or set of conditions assumed to impart the least amount of metabolic change during each year was labeled as our reference group, to which all other conditions were compared for that sample year. For Year 1 (2013), we designated “ambient” as our reference category, while samples grown under ambient temperature and ambient precipitation were designated as our reference category for Year 2 (2014). We designated samples collected from cold region, low-light conditions as our reference category for Year 3 (2015). To help visualize how different abiotic conditions may influence PSM production in different species, we calculated the number of chemical features that increased and decreased by ≥ 75%, relative to our reference category and created scaled Venn Diagrams representing these relationships.

Finally, we used linear mixed-effects models to test the main effect of abiotic condition on the relative abundance of our example compounds, with sample block as our random effect for experimental samples and plot ID as our random effect for observational samples (*lme* function within the *nlme* package, Pinheiro et al., 2015). These models tested whether combinations of abiotic factors influence the abundance of our known example compounds.

## Results

### Temperature

**FIGURE 2:**
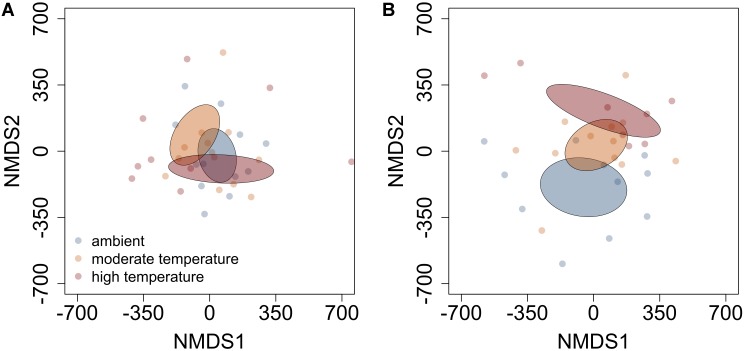
Non-metric multidimensional scaling (NMDS) plots detailing the influence of moderate and high-temperature on PSM profiles of **(A)** balsam fir and **(B)** paper birch in closed overstory. Ellipses represent 95% confidence intervals, based on standard error. In balsam fir, both warming treatments exhibit less overlap with each other than with ambient. In paper birch, different temperatures lead to distinct profiles when compared to each other and ambient.

**FIGURE 3:**
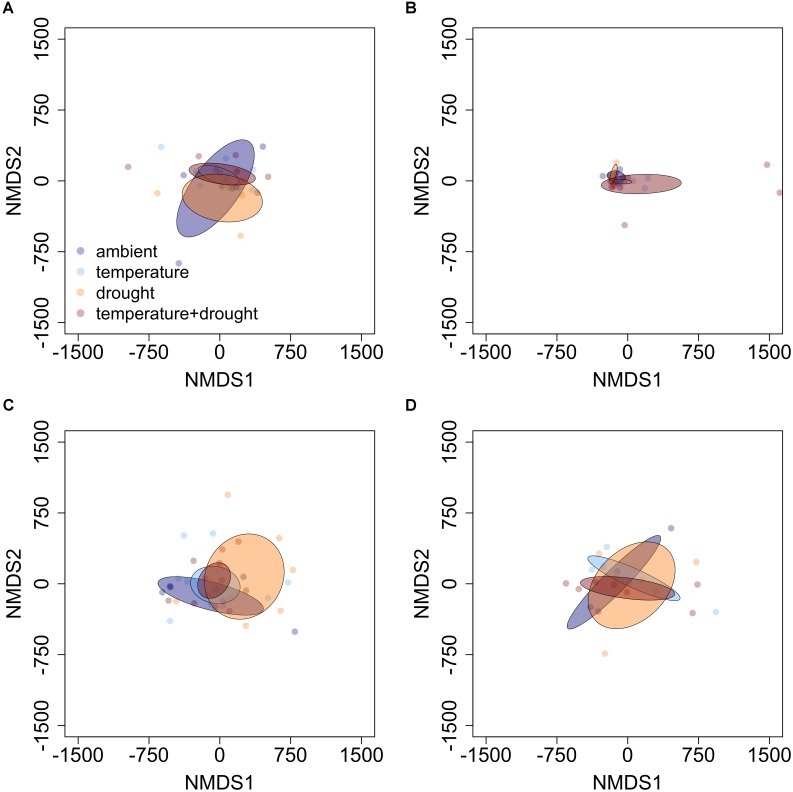
Non-metric multidimensional scaling (NMDS) plots detailing the influence of elevated temperature and drought on PSM profiles of **(A)** balsam fir, **(B)** red maple, **(C)** paper birch, and **(D)** trembling aspen in open overstory. Ellipses represent 95% confidence intervals, based on standard error. There appears to be no discernible pattern between sets of abiotic factors and PSM profiles, regardless of species.

**FIGURE 4:**
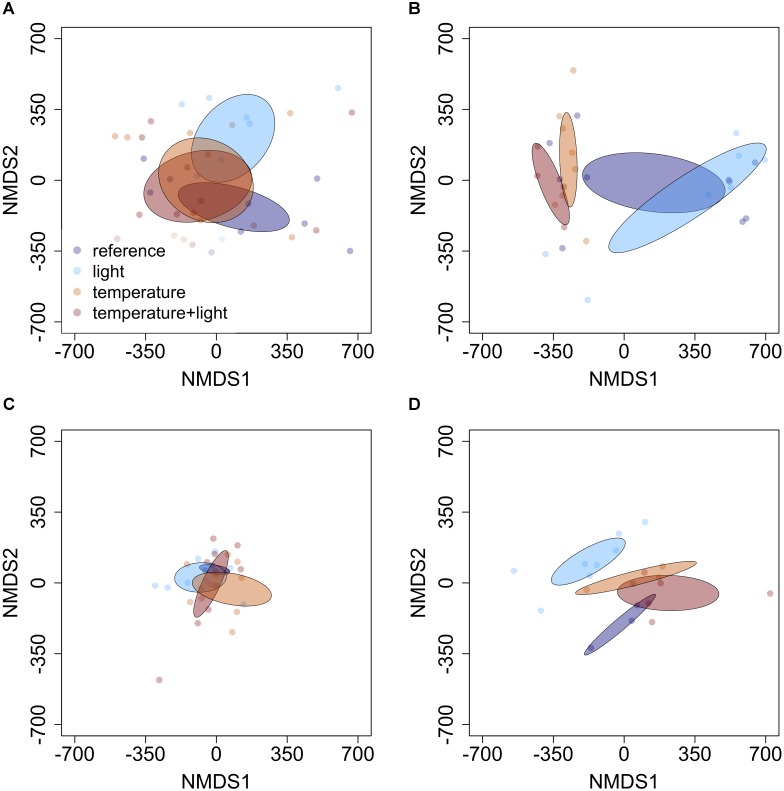
Non-metric multidimensional scaling (NMDS) plots detailing the influence of varying light and temperature conditions on PSM profiles of **(A)** balsam fir, **(B)** paper birch, **(C)** beaked hazel, and **(D)** trembling aspen. Ellipses represent 95% confidence intervals, based on standard error. Each species appears to respond to different abiotic conditions in a unique manner. Balsam fir appears to create unique PSM profiles in high-light conditions when compared to our reference group (closed canopy, low temperature), while paper birch and trembling aspen appear to have distinct PSM profiles for each set of conditions. Beaked hazel exhibits no discernible pattern.

The influence of temperature was both species and context dependent. In closed overstory (Year 1), when compared to ambient, warming-induced changes to the phytochemical profile of balsam fir were not statistically significant, whereas paper birch exhibited warming-induced shifts to phytochemical profiles, thereby leading to distinct PSM profiles for the warming treatment. Analysis of multivariate dispersion and mean-dissimilarity matrices both suggest that differences in paper birch were due to temperature-induced changes in the centroid rather than dispersion (**Table 1**). NMDS plots reveal minor overlap between temperature conditions in paper birch, and balsam fir grown under moderate and high-temperatures show strong overlap with plants grown in ambient temperatures but minor overlap with each other (**Figure 2**). Warming had no effect on phytochemical richness in either species but did influence phytochemical turnover in paper birch (**Table 1**). In open overstory (Year 2), warming had no influence on PSM profiles or PSM diversity (i.e., richness or turnover), regardless of species (**Table 1**). NMDS plots support these findings in that there is no discernible relationship between temperature and PSM profiles, regardless of species (**Figure 3**). In observational samples collected throughout northeast Minnesota (Year 3), temperature on its own had no influence on plant PSM profiles or phytochemical richness values. However, phytochemical turnover was significantly different in plants from different temperature regions in paper birch (perMANOVA, *F* = 5.912, *r*^2^ = 0.179, *P* = 0.0003) and trembling aspen (perMANOVA, *F* = 3.322, *r*^2^ = 0.156, *P* = 0.0012). NMDS plots suggest that each species responds differently to combinations of temperature and light (i.e., canopy; **Figure 4**). Balsam fir produces distinct PSM profiles as a function of ambient light conditions (i.e., open vs. closed canopy), but only within the cool region, while paper birch and trembling aspen appear to have distinct PSM profiles for each combination of conditions. Conversely, beaked hazel exhibits no discernible pattern across different conditions.

**Table 1:** Summary of results for B4WarmED samples used to assess the influences of temperature and drought on PSM profiles and phytochemical diversity.

Year	Species	Stress condition	*n*	Features	PSM profile						Phytochemical diversity				
					perMANOVA			Dispersion		Centroid	LME_richness_		perMANOVA_turnover_		
					*F*	*r^2^*	*P*	*F*	*P*	Δ	Δ_richness_	*P*	*F*	*r^2^*	*P*
2013	Balsam fir	Ambient^a^	12	1903	1.223	0.073	0.103	0.576	0.567	na	na	na	1.206	0.072	0.142
		Mod. temp.	13	1856						–25.800	–47	0.154			
		High temp.	9	1873						–68.500	–30	0.321			
	Paper birch	Ambient^a^	11	1669	1.382	0.090	*0.013^∗^*	0.765	0.470	na	na	na	1.444	0.093	*0.019^∗^*
		Mod. temp.	12	1722						55.700	53	0.201			
		High temp.	8	1700						17.700	31	0.526			
2014	Balsam fir	Ambient^a^	5	1937	1.016	0.105	0.428	0.346	0.810	na	na	na	1.076	0.110	0.308
		Temp.	11	2017						196.000	80	0.222			
		Drought	5	2012						121.000	75	0.308			
		Temp. + drought	9	1992						118.000	55	0.308			
	Red maple	Ambient^a^	5	1968	1.070	0.100	0.303	1.520	0.210	na	na	na	1.076	0.100	0.320
		Temp.	11	2002						29.300	34	0.800			
		Drought	4	1998						97.600	30	0.857			
		Temp. + drought	13	1845						–251.300	–123	0.344			
	Paper birch	Ambient^a^	6	1948	1.149	0.097	0.147	1.233	0.307	na	na	na	1.210	0.102	0.134
		Temp.	12	2014						32.000	66	0.232			
		Drought	7	1949						–112.000	1	0.973			
		Temp. + drought	11	2036						98.000	88	0.122			
	Trembling aspen	Ambient^a^	4	2287	0.689	0.103	0.960	0.061	0.980	na	na	na	0.622	0.094	0.980
		Temp.	6	2282						17.000	–5	0.961			
		Drought	5	2241						–44.000	–46	0.646			
		Temp. + drought	7	2282						16.000	–5	0.957			


*For samples collected during 2013, “mod. temp.” includes all samples collected from plots warmed to ambient + 1.7°C, while “high temp.” includes all samples collected from plots warmed to ambient + 3.4°C. For a given stress condition, the mean number of chemical features identified within a species is listed under “features.” “Dispersion” represents the results of our multivariate homogeneity of variance test, while “centroid” represents the mean difference in dissimilarity matrices relative to our reference group (^∗^). A larger Δ value indicates greater distance from the reference group than those with a smaller Δ. All statistical analyses were tested against α = 0.05, and statistically significant results are italicized and identified with an asterisk (^∗^). ^*a*^Reference group or baseline condition for the given sample year to which all other treatments within species were compared. na indicates not applicable.*

Venn diagrams created to help visualize the influence of different abiotic conditions for Year 1 samples suggest that the high-temperature (+3.4°C) treatment induced a greater response from both balsam fir and paper birch than the moderate-temperature (+1.7°C) treatment. Specifically, the high-temperature treatment led to more features that either increased or decreased in relative abundance by 75% or more when compared to ambient or moderate-temperature treatments (**Table 2** and **Supplementary Figures S4–S6**).

**Table 2:** Number of chemical features that increase and decrease in relative abundance by ≥ 75% as a function the dominant stress condition.

Year	Species	Increase by ≥ 75%		Decrease by ≥ 75%	
		Stress condition	Number affected	Stress condition	Number affected
2013	Balsam fir	High Temperature	6	High Temperature	21
	Paper birch	High Temperature	28	High Temperature	38
2014	Balsam fir	Drought	43	Temperature + Drought	35
	Paper birch	Drought	98	Temperature + Drought	31
	Red maple	Temperature + Drought	36	Drought	66
	Trembling aspen	Temperature	79	Drought	37
2015	Balsam fir	Temperature + Light	26	Light	111
	Beaked hazel	Temperature + Light	155	Temperature + Light	56
	Paper birch	Temperature + Light	126	Light	278
	Trembling aspen	Temperature + Light	280	Light	162


*In most scenarios, the stress condition that led to large-scale increases in relative abundance was different than that which led to large-scale decreases. “Number affected” displays the number of chemical features that either increased or decreased by ≥ 75% for the given species and stress condition.*

### Interactive Effects of Different Abiotic Conditions

In our Year 2 samples, the combination of drought and elevated temperature had no influence on PSM profiles or any aspect of phytochemical diversity, regardless of species (**Table 1**). These results were supported by NMDS plots (**Figure 3**). Additionally, Venn diagrams suggest large-magnitude increases or decreases in relative abundance of PSMs did not follow an obvious pattern that could be attributed to different conditions. There appears to be a high degree of overlap across conditions in those compounds that exhibit increases in relative abundance of ≥ 75%, while less overlap occurs among compounds exhibiting large declines in relative abundance. Furthermore, the influence of drought on the decline of relative abundance by ≥ 75% appears to be more distinct than that of either warming or warming and drought together (**Table 2** and **Supplementary Figures S4–S6**).

In observational samples from throughout northeast Minnesota (Year 3), when evaluating the effects of high temperature and light combined, balsam fir appears to create unique PSM profiles in response to different light conditions (i.e., open vs. closed canopy), but only within the cool region, while paper birch and trembling aspen appear to have distinct PSM profiles for each condition. Beaked hazel exhibits no discernible pattern (**Figure 4**). Phytochemical richness did not vary as a function of light conditions or temperature region. However, phytochemical turnover in balsam fir was significantly influenced by conditions of high light (i.e., open canopy; **Table 3**). When analyzing the interactive effects of light conditions and temperature region, all species exhibited significant differences in their PSM profile (**Table 3**), with only trembling aspen exhibiting significant differences in multivariate dispersion as a function of the combination of light condition and temperature region (**Table 3**). Although phytochemical richness was not influenced by the combined effects of temperature region and light conditions, phytochemical turnover was influenced in paper birch and trembling aspen and a marginal, non-significant trend was present in beaked hazel (**Table 3**).

**Table 3:** Summary of results for observational samples used to assess the influences of temperature region and overstory on PSM profiles and phytochemical diversity.

Year	Species	Stress condition	*n*	Features	PSM profile						Phytochemical diversity				
					perMANOVA			Dispersion		Centroid	LME_richness_		perMANOVA_turnover_		
					*F*	*r^2^*	*P*	*F*	*P*	Δ	Δ_richness_	*P*	*F*	*r^2^*	*P*
2015	Balsam fir	Reference^a^	10	1371	1.579	0.119	*0.024^∗^*	0.334	0.807	na	na	na	2.152	0.156	*0.004^∗^*
		Light	8	1287						27.1	–84	0.228			
		Temp.	10	1373						–11.8	2	0.947			
		Temp. + light	11	1361						–40.1	–10	0.844			
	Paper birch	Reference^a^	10	1185	2.029	0.196	*0.002^∗^*	2.546	0.072	na	na	na	2.784	0.250	*0.001^∗^*
		Light	7	1168						–2.5	–17	0.675			
		Temp.	8	1205						88.5	20	0.708			
		Temp. + light	4	1223						143.4	38	0.537			
	Beaked hazel	Reference^a^	3	1338	1.968	0.269	<*0.001^∗^*	0.242	0.863	na	na	na	1.313	0.120	0.109
		Light	8	1220						–227.8	–118	0.467			
		Temp.	12	1194						–262.1	–144	0.303			
		Temp. + light	10	1252						–228.1	–86	0.546			
	Trembling aspen	Reference^a^	3	1509	1.352	0.123	*0.028^∗^*	2.92	*0.040^∗^*	na	na	na	2.696	0.336	<*0.001^∗^*
		Light	8	1466						–26.2	–43	0.556			
		Temp.	3	1531						–23.8	22	0.789			
		Temp. + light	6	1558						–36.4	49	0.537			


*For a given condition, the mean number of chemical features identified within a species is listed under “features.” “Dispersion” represents the results of our multivariate homogeneity of variance test, while “centroid” represents the mean difference in dissimilarity matrices relative to our reference group (^∗^). A larger Δ value indicates greater distance from the reference group than those with a smaller Δ. All statistical analyses were tested against α = 0.05, and statistically significant results are italicized and identified with an asterisk (^∗^). ^*a*^Reference group or baseline condition (i.e., lower temperatures, low light) to which all other treatments were compared. na indicates not applicable.*

Patterns in Venn diagrams detailing the influences of different conditions during Year 2 are difficult to discern, as different plant species appeared to respond to varying conditions in different ways (**Table 2** and **Supplementary Figure S5**). Drought led to more features increasing by ≥ 75% in balsam fir and paper birch, while elevated temperature led to the large-magnitude increase of more features in trembling aspen (**Table 2** and **Supplementary Figure S5**). In red maple, the combination of drought and elevated temperature had the greatest influence on large-magnitude increases in relative abundance (**Table 2** and **Supplementary Figure S5**). The combination of drought and warming led to more large-magnitude declines in relative abundance in balsam fir and paper birch, while drought had a greater impact on red maple and trembling aspen (**Table 2** and **Supplementary Figure S5**). In observational samples (Year 3), the combination of high-light conditions and warmer temperatures led to more large-magnitude shifts in relative abundance (i.e., increasing and decreasing by 75% or more), regardless of species (**Table 2** and **Supplementary Figure S6**).

### Example Compounds

**FIGURE 5:**
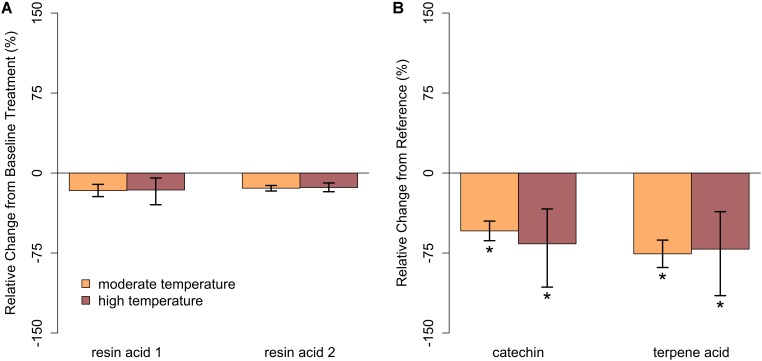
Relative change in abundance (%) for specific PSM compounds when compared to our reference treatment for Year 1 (ambient temperature) for **(A)** balsam fir and **(B)** paper birch in closed overstory. Neither resin acid in balsam fir was influenced by warming. In paper birch, both catechin and terpene acid declined with warming relative to ambient. Error bars represent the 95% boot-strapped confidence intervals and relative abundances significantly different than those found in the baseline treatment are identified by an asterisk (^∗^).

**FIGURE 6:**
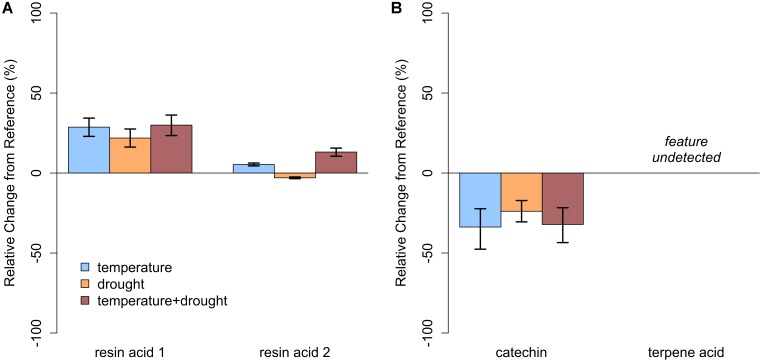
Relative change in abundance (%) for specific PSM compounds when compared to our baseline treatment for Year 2 (ambient temperature, ambient precipitation) for **(A)** balsam fir and **(B)** paper birch in open overstory. Neither resin acid in balsam fir was influenced by warming. In paper birch, relative abundance of catechin was not influenced by temperature; however, terpene acid was undetected. Error bars represent the 95% boot-strapped confidence intervals.

**FIGURE 7:**
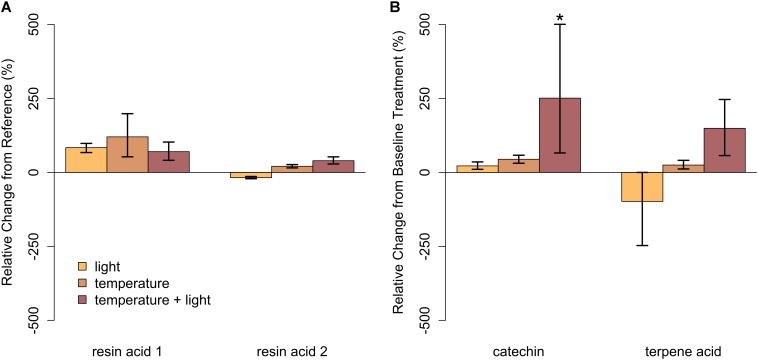
Relative change in abundance (%) for specific PSM compounds when compared to our baseline treatment for Year 3 (cold region, closed overstory) for **(A)** balsam fir and **(B)** paper birch. Neither resin acid in balsam fir was influenced by warming. In paper birch, relative abundance of catechin was only influenced by the combination of light and high temperatures, increasing by more than 250%. Terpene acid was unaffected, regardless of stress condition. Error bars represent the 95% boot-strapped confidence intervals and relative abundances significantly different than those found in the reference condition are identified by an asterisk (^∗^).

In closed-overstory conditions (Year 1), warming resulted in significant declines in both catechin and terpene acid in paper birch but had no influence on either compound in balsam fir (**Figure 5** and **Supplementary Table S3**). In high-light conditions (Year 2), neither of the compounds in either species exhibited a significant, condition-specific change in relative abundance. However, terpene acid in paper birch was completely absent from all samples collected from high-light plots (**Figure 6** and **Supplementary Table S3**). In observational samples from throughout northeast Minnesota (Year 3), neither compound in balsam fir exhibited significant changes in relative abundance due to light conditions, temperature region, or their interaction. In paper birch, however, the interactive effects of high-light conditions and warmer-temperatures resulted in a more than 250% increase in the relative abundance of catechin, while terpene acid exhibited no response, regardless of treatment (**Figure 7** and **Supplementary Table S3**).

## Discussion

Our study is among the first to explicitly show that combinations of abiotic drivers (often potential stressors) in forest plants can lead to broad phytochemical responses that are distinct from those that result from single abiotic factors and that different species of woody plants respond to complex sets of conditions in variable ways. In our experimental samples, warming under closed canopy led to distinct PSM profiles in paper birch but not balsam fir, with paper birch exhibiting increased phytochemical turnover. Warming under open canopy had no influence on PSM profiles or any aspect of phytochemical diversity. In our observational samples collected across northeast Minnesota, warmer temperatures had no influence on PSM profiles but did lead to significant phytochemical turnover in paper birch and trembling aspen. When elevated temperature was combined with drought in Year 2 of our experimental samples, we found no influence on PSM profiles or phytochemical diversity. However, temperature variation combined with high-light conditions in our observational samples resulted in condition-specific profiles for all species and led to significant phytochemical turnover in all but beaked hazel. In general, our results indicate that the phytochemical response of plants to varying combinations of abiotic factors cannot be directly extrapolated from the response of plants to individual factors. Perhaps more importantly, our results provide evidence that heterogeneity in the abiotic environment influences secondary metabolism in woody plants via a range of complex and highly variable responses.

Few studies to date have explicitly studied the influences of heterogeneity in the abiotic environment on phytochemical diversity, and specifically, phytochemical turnover. However, it has been hypothesized that variability in which compounds are either present or absent may be an adaptation for variable environments, thereby decreasing vulnerability of plants to a range of potential stress conditions, including herbivory (Laitinen et al., 2000; Cheng et al., 2011). Here, we found that in some plants species, different combinations of abiotic factors can affect which compounds are either present or absent, thus leading to phytochemical turnover. For example, compounds that are absent in one set of conditions may become present within a slightly different set of conditions, or vice versa. The potential for this to occur was apparent when our example terpene acid decreased in paper birch plants subjected to experimentally elevated temperature in closed canopy but went completely undetected in plants subjected to experimental warming and drought in open canopy and exhibited no change at all in our observational samples from throughout northeast Minnesota. Suppression of individual compounds due to varying stress conditions has been observed in other studies as well. For instance, proline, which is thought to play an important role in protection from drought, is severely suppressed when plants are simultaneously subjected to drought and high temperatures (Rizhsky et al., 2004). While individual compounds can play an important role in the survival of plants subjected to a range of biotic and abiotic conditions, a plant’s phytochemical profile imparts a metabolic framework that can determine the biological role and strength of individual compounds (Dyer et al., 2003; Richards et al., 2010; Gershenzon et al., 2012; Jamieson et al., 2015). Here, we show that individual compounds as well as the phytochemical context within which they operate can both be altered by variations in the abiotic environment.

Plants produce thousands of individual compounds, and variations in the relative abundance of many of these compounds can have a wide-range of effects on the biotic interactions plants have with other organisms. Catechin, which is a phenol-based precursor to proanthocyanidins (i.e., condensed tannins), is widely considered an antiherbivore defensive compound (Tahvanainen et al., 1985; Berg, 2003; Stolter et al., 2005) and can have a significant, negative impact on the development of forest pests (Roitto et al., 2009). Catechin also has antimicrobial and allelopathic effects, and plants with decreased catechin production may be at a competitive disadvantage for nutrients within the soil as it can inhibit the growth and germination of neighboring plants (Veluri et al., 2004; Inderjit et al., 2008). Terpene acids, including diterpene resin acids, are considered strong antifeedants (Ikeda et al., 1977), and the ingestion of forage with elevated concentrations of diterpenoids can result in slower development times and significantly higher mortality in herbivorous larvae (Larsson et al., 1986). Here, we show that different compounds have individualized responses based on the micro-environmental conditions that are present.

In balsam fir, warming alone led to consistent, albeit non-significant declines in the mean relative abundances of both resin acids. When high temperatures were combined with other abiotic factors (i.e., drought and light), resin acid 1 exhibited consistent but non-significant increases, while resin acid 2 was more variable, exhibiting no consistent trend. In paper birch, both example compounds exhibited significant changes in relative abundance as a function of different factors. While elevated temperature alone led to significant declines in catechin, the combination of elevated temperature and high light led to a more than 250% increase in relative abundance. Our example terpene acid declined with warming and was undetectable when we tried to assess the effects of drought. This particular scenario provides an example of how individual compounds may “wink in or out” due to variation in the abiotic environment.

Numerous studies have reported that high-temperature and drought interact to alter PSM production in plants (Craufurd and Peacock, 1993; Savin and Nicolas, 1996; Jiang and Huang, 2001; Rizhsky et al., 2002, 2004). Thus, we were surprised to find no interaction between drought and warming in our study. It is important to note, however, that the extremes of those treatments employed by other studies are typically greater than what we test here, sometimes increasing temperature to more than 40°C (Rizhsky et al., 2002) and withholding water altogether for extended periods (Jiang and Huang, 2001). In our study, mean soil moisture was lower during 2014 than 2013, with mean soil temperatures being higher (unpublished data). Surprisingly, air temperature and leaf-tissue surface temperature during late spring/early summer (May 1 to July 15) were indistinguishable between samples years and plot types (2013 closed canopy vs. 2014 open canopy), and cumulative precipitation during the first half of each year (January 1 to July 15) was also indistinguishable (unpublished results). Combinations of abiotic factors can have one dominant factor that defines the phytochemical response of affected plants, and drought, when present, may dominate the influence of combinations of abiotic factors. Considering this, our inability to identify any treatment-specific influence on PSM profiles or phytochemical diversity may be due to low soil moisture during 2014. If plants from which samples were collected from in 2014 were experiencing some level of drought stress due to low soil moisture, this signal may have preempted any potential phytochemical response that might have occurred due to treatment.

When considering the influence of abiotic conditions on large-scale shifts in relative abundance (increases or decreases ≥ 75% relative to our reference group), greater increases in temperature (+3.4°C) appeared to have a greater influence than moderate increases (+1.7°C). When present, drought, either alone or in combination with elevated temperature, dominated all but one of the large-scale shifts we assessed (Year 2), and in our observational samples, high-light conditions, either alone or in combination with elevated temperature, dominated all of the large-scale shifts we assessed in which it was present (Year 3). As noted above, numerous studies have reported that drought has a defining impact on plants’ phytochemical profiles, even when in combination with other abiotic drivers, such as elevated temperature and high light. Moreover, in our Year 1 samples, elevated temperature led to both large-scale increases and large-scale decreases in relative abundance. However, the number of compounds exhibiting these shifts was substantially smaller when compared to the number of compounds influenced by the abiotic conditions evaluated in either Year 2 of our experimental samples or our observational samples (Year 3). Outside of Year 1, during which we tested only the effects of elevated temperature, it was rare when the same abiotic condition simultaneously dominated both large-scale increases and large-scale decreases in relative abundance, suggesting that different combinations of abiotic factors may influence upregulation and downregulation of different compounds.

Changes in the abundance and diversity of secondary metabolites within a plant’s phytochemical profile may alter biotic interactions, potentially leading to broad-scale ecological change. For example, while some herbivores respond negatively to forage with a higher diversity of PSMs, others appear to target these plants in an effort to alleviate costs associated with external stressors via their pharmacological benefits (Forbey and Hunter, 2012). Additionally, numerous studies have reported that phytochemical diversity within a plant community is positively correlated with community diversity across multiple trophic levels (Jones and Lawton, 1991; Richards et al., 2015), influencing invertebrate predators and parasitoids, and potentially extending to vertebrate predators as well (Dicke et al., 2012).

While the consequences of different abiotic conditions on phytochemical diversity remain unpredictable, our results demonstrate that the phytochemical response of plants to combinations of abiotic factors cannot be extrapolated from that of individual factors. For instance, while warming alone may have a very specific influence on some compounds, when in combination with additional abiotic factors such as drought and light, warming may lead to highly variable and unpredictable response (Mittler, 2006), making it increasingly difficult to predict the performance of woody plants in a changing environment. Regardless, previous research suggests that changes in phytochemical production induced by variability in abiotic conditions can influence both tree resistance and pest performance traits (Jamieson et al., 2015), potentially altering the frequency and intensity of insect outbreaks (Schwartzberg et al., 2014). Elevated temperatures by themselves have been shown to reduce the competitive abilities of more southern boreal tree species when compared to co-occurring species adapted to warmer climates (Reich et al., 2015). Climate-induced changes to phytochemistry may lead to shifts in the competitive landscapes for cold-adapted trees and shrubs, potentially altering their ability to compete for resources and defend against pests and pathogens in novel climatic conditions. However, because individual compounds and the metabolic profiles of which they are a part are differentially influenced by abiotic factors and combinations of these factors, predicting how forest plants will respond to novel environmental conditions will be challenging.

The majority of biotic interactions between plants and other organisms are chemically mediated, and recent climate change has challenged our understanding of the mechanisms underlying these interactions. The primary objective of this study was to determine how warming influences plant production of secondary metabolites and how combinations of additional abiotic factors may modulate this effect. Here, we show that heterogeneity in a range of abiotic factors broadly influence secondary chemistry in plants thereby leading to condition-specific phytochemical profiles. If our results are typical of plant responses, abiotically induced changes to secondary chemistry in woody plants could influence their rate of range expansion or contraction under novel climate scenarios. Additionally, our results contribute to current efforts to understand how continued warming will influence plants and the biotic interactions that serve as the foundation for a wide range of ecosystem processes. In the future, studies monitoring physiological changes in conjunction with global shifts in PSM profiles would provide insights into mechanisms underlying biotic interactions mediated by the local environment. As spatial and temporal patterns in the global abiotic environment continue to shift, it is imperative that we continue to learn as much as we can about the phytochemical response of plants to these changes.

## Author Contributions

JB, SB, AH, RaM, ReM, and JF formulated the study idea and developed the study methods while PR and ReM established the experimental study sites critical for the execution of this study. JB performed all the sample collection, while JB and SB performed the analytical chemistry and pre-statistical data processing. JB, RaM, and JF statistically analyzed the data. JB and SB wrote the initial draft of the manuscript. All the authors contributed to the manuscript revisions and approved the final manuscript.

## Conflict of Interest Statement

The authors declare that the research was conducted in the absence of any commercial or financial relationships that could be construed as a potential conflict of interest.
