# Supplementary material for: Combinations of Abiotic Factors Differentially Alter Production of Plant Secondary Metabolites in Five Woody Plant Species in the Boreal-Temperate Transition Zone
Source: Front Plant Sci. 2018 Sep 5;9:1257. doi: 10.3389/fpls.2018.01257 (PMC6134262; doi:10.3389/fpls.2018.01257)
Supplement: Supplementary file 1 [file Table_1.pdf]

Table S1. Location and treatment data of field sampling locations. Light conditions were a binary response of whether a plot was in an area that was recently clear cut (i.e., high) or areas that have experienced no known overstory disturbance since at least 1985 (i.e., low). Mean-maximum summer temperature (MMST) is the maximum daily temperature averaged across June, July, and August from 1981 to 2000 (PRISM Climate Group, 2017).

plot ID	easting	northing	light conditions	MMST
1	700598	5303917	high	23.86
2	700283	5308537	high	25.30
3	698786	5308655	high	25.05
4	685385	5304339	low	24.70
5	685160	5295714	low	22.86
6	678345	5291770	low	22.36
7	614050	5313377	low	25.40
8	611435	5315050	low	25.62
9	610716	5314932	low	25.68
10	599534	5312110	high	25.51
11	599427	5312111	high	25.51
12	598718	5310922	high	25.43

Table S2. Experimental exact mass, hypothetical mass, molecular formula and PPM error for all metabolites found to be significantly abundant (ANOVA, $\alpha = 0.001$). "Level of Confidence" signifies the level of confidence in metabolite 'identification', as defined by the Chemical Analysis Working Group of the Metabolomics Standards Initiative (Sumner et al., 2007). Catechin was found to be significant in both positive and negative ionization modes, and its identity was confirmed via an authentic standard.

Identification	Species	Ionization mode	Experimental exact mass	Hypothetical exact mass	Molecular formula	PPM error	Level of Confidence
catechin	*paper birch*	-	289.0729	289.0712	$C_{15}H_{13}O_6$	5.8809	1
catechin	*paper birch*	+	291.0861	291.0868	$C_{15}H_{15}O_6$	2.4048	1
putative diterpene resin acid 1	*balsam fir*	+	317.1382	317.1389	$C_{18}H_{21}O_5$	2.2072	3
putative diterpene resin acid 2	*balsam fir*	+	331.1541	331.1545	$C_{19}H_{23}O_5$	1.2079	3
putative diterpene resin acid 3	*paper birch*	+	337.1435	337.1439	$C_{21}H_{21}O_4$	1.1864	3

Table S3. Results of linear mixed-effects models comparing changes in relative abundance of example compounds for different stress conditions. Statistically significant results ($\alpha = 0.05$) are identified with an asterisk (*) and change values preceded by "–" indicate a decline in mean relative abundance relative to our reference group, where as a "+" indicates an increase in mean relative abundance.

species	compound	year	stress condition	df	change (%)	t	P
balsam fir	resin acid 1	1	moderate temperature	29	-16.5	-0.724	0.4751
		1	high temperature	29	-15.9	-0.372	0.7126
		2	drought	25	+21.9	0.795	0.4343
		2	temperature	25	+28.7	1.238	0.2272
		2	drought + temperature	25	+30.0	1.234	0.2288
		3	light	8	+83.7	0.977	0.3570
		3	temperature	8	+120.2	1.484	0.1761
		3	temperature + light	8	+70.2	0.878	0.4057
	resin acid 2	1	moderate temperature	29	-14.2	-1.068	0.2944
		1	high temperature	29	-13.6	-0.919	0.3657
		2	drought	25	-3.0	-0.103	0.9188
		2	temperature	25	+5.4	0.333	0.7417
		2	drought + temperature	25	+13.1	0.736	0.4685
		3	light	8	-17.9	-0.586	0.5739
		3	temperature	8	+20.8	0.815	0.4389
		3	temperature + light	8	+39.5	1.495	0.1734
paper birch	catechin	1	moderate temperature	26	-54.3	-3.933	*< 0.0001**
		1	high temperature	26	-66.4	-4.322	*0.0002**
		2	drought	30	-23.9	-1.070	0.2931
		2	temperature	30	-33.8	-1.618	0.1161
		2	drought + temperature	30	-32.2	-1.489	0.1469
		3	light	6	+22.0	0.310	0.7670
		3	temperature	6	+44.5	0.626	0.5538
		3	temperature + light	6	+251.1	2.837	*0.0297**
	terpene acid	1	moderate temperature	26	-75.8	-3.015	*0.0057**
		1	high temperature	26	-71.4	-2.877	*0.0079**
		2	drought				
		2	temperature		*feature undetected*		
		2	drought + temperature				
		3	light	6	-98.0	-1.504	0.1832
		3	temperature	6	24.9	0.283	0.7870
		3	temperature + light	6	149.1	1.664	0.1470

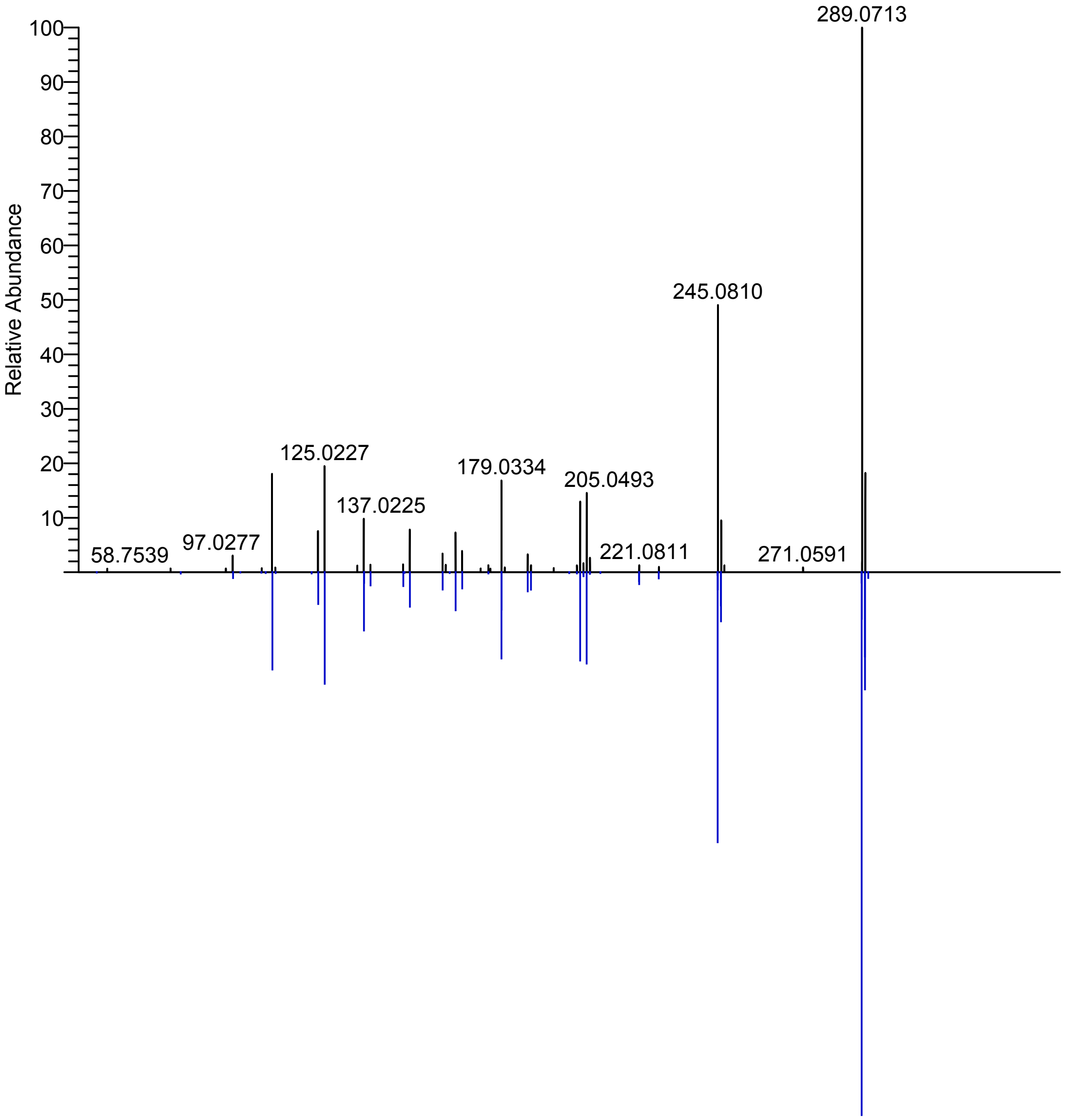

Figure S1. Mirrored HCD fragmentation spectra of endogenous catechin from Year 1 paper birch (above) and a catechin standard (below) from negative ionization mode. Catechin was identified and shown to be distinct from its isomer, epicatechin, as commercial standards of each of these compounds were chromatographically resolved. HCD fragmentation was performed at a normalized collision energy of 25.

(A)

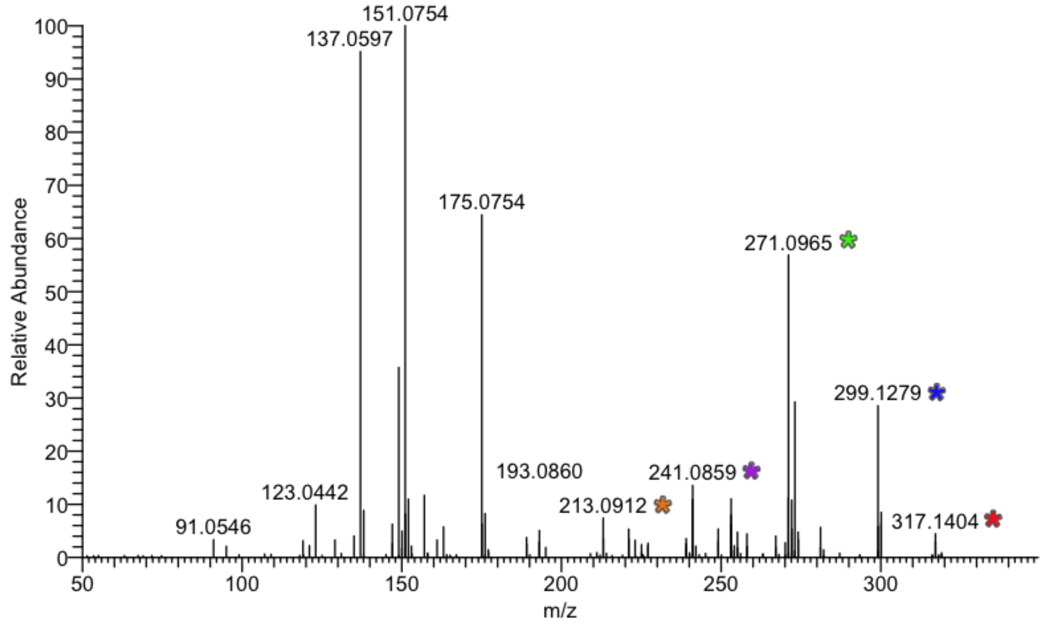

(B)

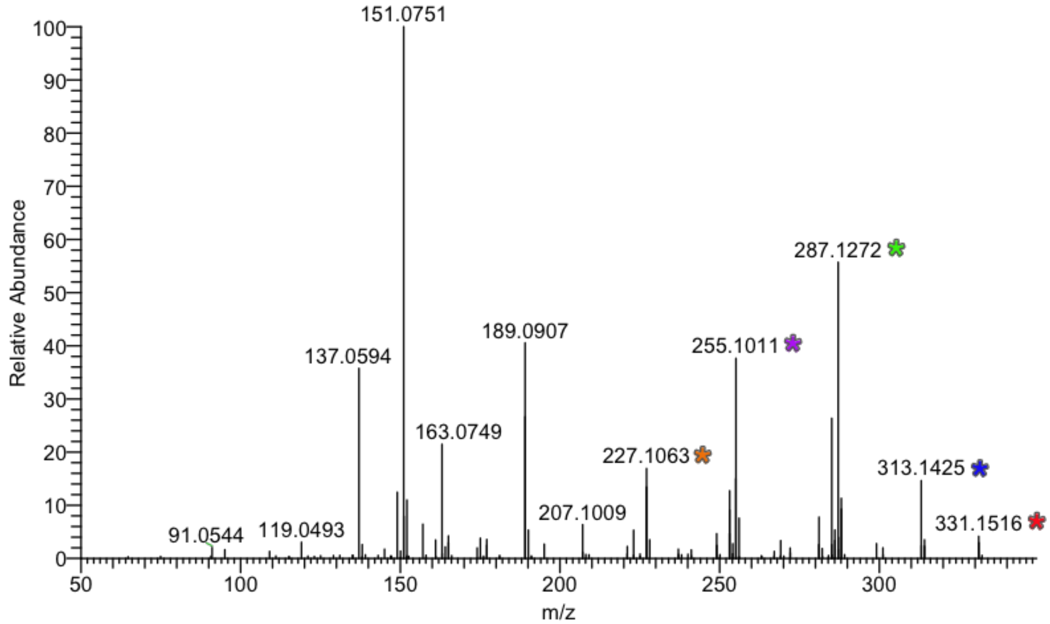

Figure S2. Positive ionization mode HCD fragmentation spectra of putative diterpene resin acids with *m/z* values of 317.1382 (A) and 331.1542 (B) from balsam fir. HCD fragmentation was performed at a normalized collision energy of 10. The spectra are very similar except for a 14 AMU shift (denoted with a *), suggesting these molecules are structurally related and differ only in the length of a hydrocarbon chain or presence/absence of a methylation.

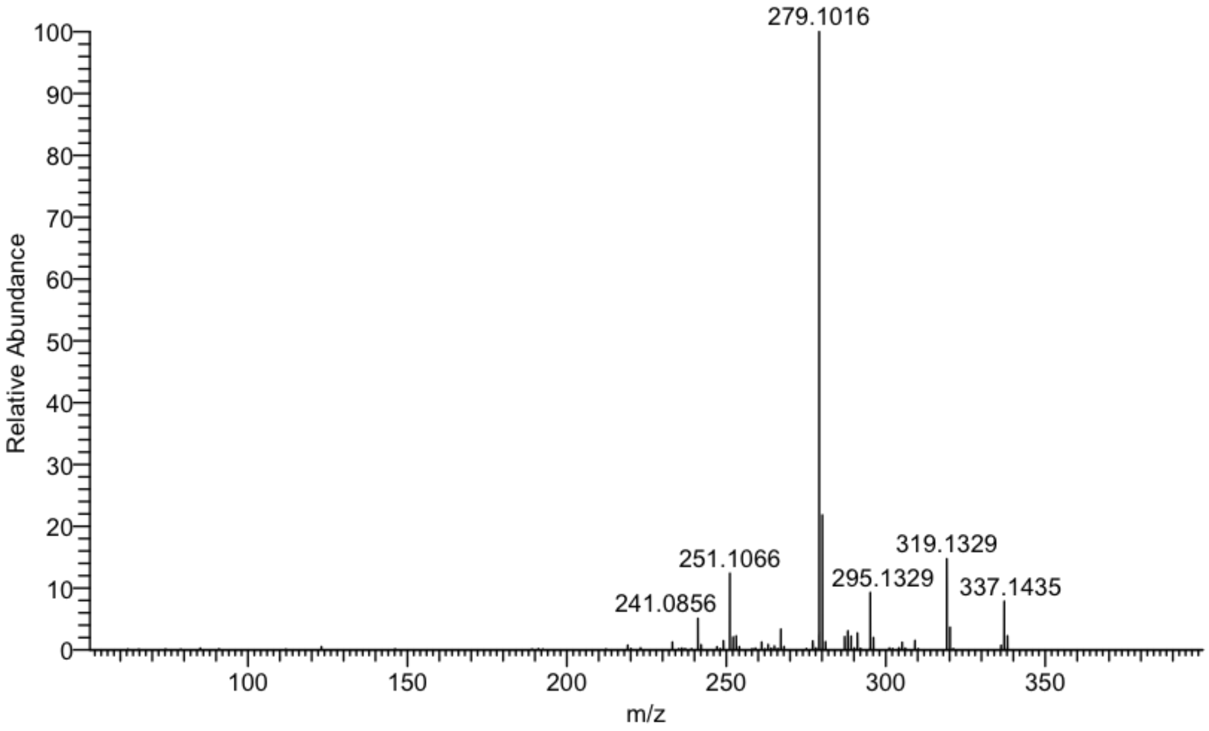

Figure S3. Positive ionization mode HCD fragmentation spectra of putative diterpene resin acid from paper birch. HCD fragmentation was performed at a normalized collision energy of 25.

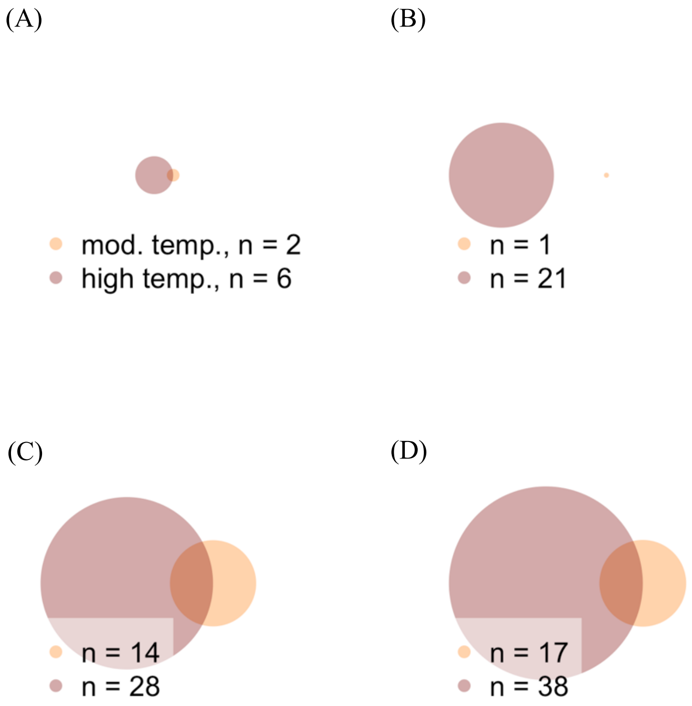

Figure S4. Venn diagrams for Year 1 samples detailing the number of compounds that increase or decrease by ≥ 75% in balsam fir (A and B, respectively) and paper birch (C and D, respectively). Circles are scaled and comparable across species and treatments. Areas in which circles are overlapping are relative to the number of compounds effected by both treatments. High-temperatures appears to have a greater influence on large scale shifts in the relative abundance of compounds than moderate temperatures.

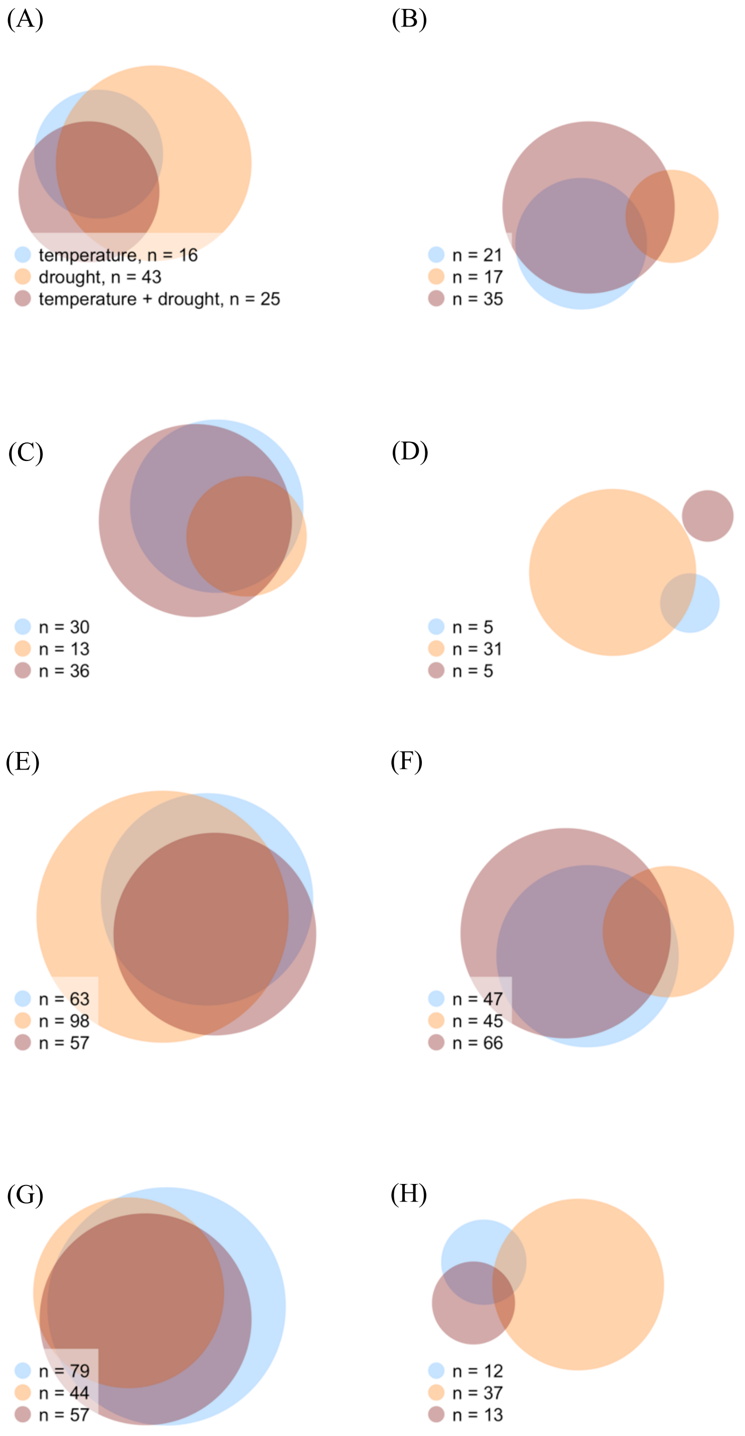

Figure S5. Venn diagrams for Year 2 samples detailing the number of compounds that increase or decrease by ≥ 75% in balsam fir (A and B, respectively), red maple (C and D, respectively), paper birch (E and F, respectively), and trembling aspen (G and H, respectively). Circles are scaled and comparable across species and treatments. Areas in which circles are overlapping are relative to the number of compounds affected by all treatments.

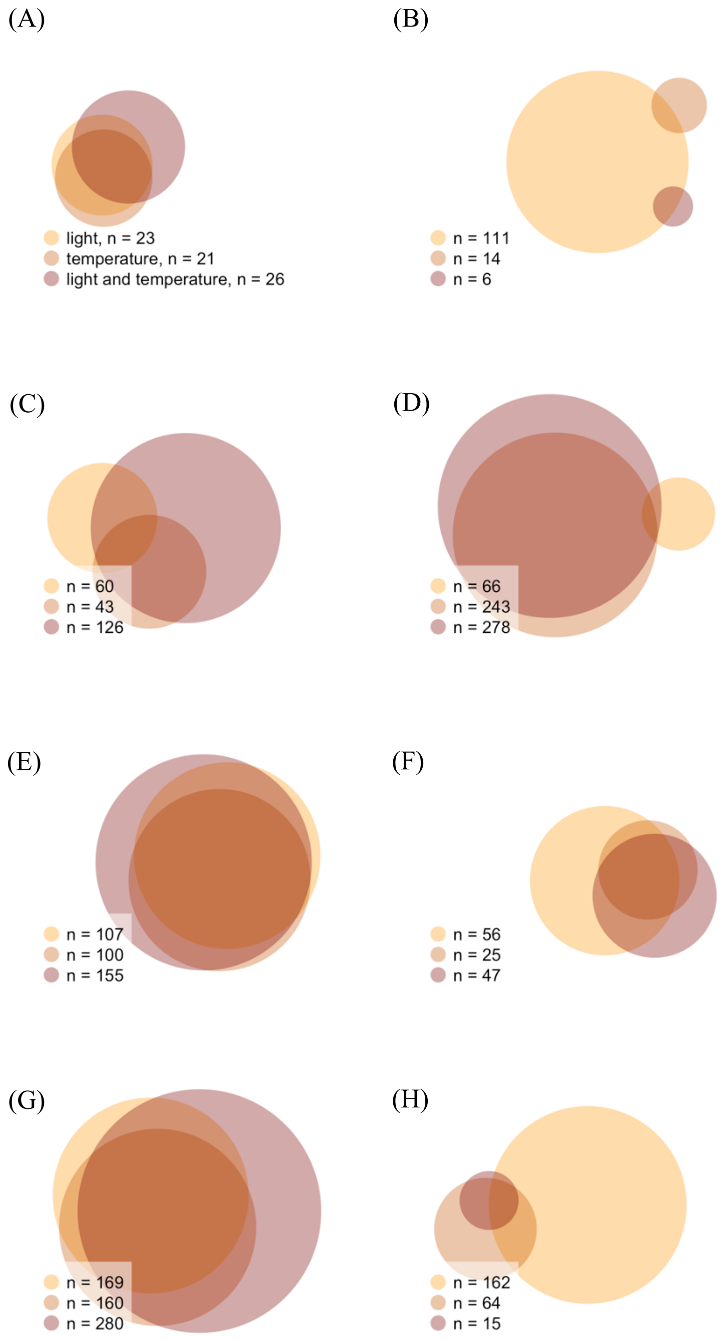

Figure S6. Venn diagrams for Year 3 samples detailing the number of compounds that increase or decrease by ≥ 75% in balsam fir (A and B, respectively), paper birch (C and D, respectively), beaked hazel (E and F, respectively), and trembling aspen (D and H, respectively). Circles are scaled and comparable across species and treatments. Areas in which circles are overlapping are relative to the number of compounds affected by all treatments. In general, the combination of high-light and high-temperature results in the large-scale increase of more compounds, on average, than any other treatment. While high-light conditions result in the large-scale decrease of more compounds, on average, than any other treatment.

References

PRISM Climate Group (2017). *PRISM Climate Group*. Available at: http://prism.oregonstate.edu.

Sumner, L. W., Amberg, A., Barrett, D., Beale, M. H., Beger, R., Daykin, C. A., et al. (2007). Proposed minimum reporting standards for chemical analysis. *Metabolomics* 3, 211–221.
