# Supplementary material for: Combinations of Abiotic Factors Differentially Alter Production of Plant Secondary Metabolites in Five Woody Plant Species in the Boreal-Temperate Transition Zone
Source: Front Plant Sci. 2018 Sep 5;9:1257. doi: 10.3389/fpls.2018.01257 (PMC6134262; doi:10.3389/fpls.2018.01257)
Supplement: Supplementary file 2 [file Table_2.pdf]

Table S2. Experimental exact mass, hypothetical mass, molecular formula and PPM error for all metabolites found to be significantly abundant (ANOVA, $\alpha = 0.001$). "Level of Confidence" signifies the level of confidence in metabolite 'identification', as defined by the Chemical Analysis Working Group of the Metabolomics Standards Initiative (Sumner et al., 2007). Catechin was found to be significant in both positive and negative ionization modes, and its identity was confirmed via an authentic standard.

Identification	Species	Ionization mode	Experimental exact mass	Hypothetical exact mass	Molecular formula	PPM error	Level of Confidence
catechin	*paper birch*	-	289.0729	289.0712	$C_{15}H_{13}O_6$	5.8809	1
catechin	*paper birch*	+	291.0861	291.0868	$C_{15}H_{15}O_6$	2.4048	1
putative diterpene resin acid 1	*balsam fir*	+	317.1382	317.1389	$C_{18}H_{21}O_5$	2.2072	3
putative diterpene resin acid 2	*balsam fir*	+	331.1541	331.1545	$C_{19}H_{23}O_5$	1.2079	3
putative diterpene resin acid 3	*paper birch*	+	337.1435	337.1439	$C_{21}H_{21}O_4$	1.1864	3
