# Supplementary material for: Combinations of Abiotic Factors Differentially Alter Production of Plant Secondary Metabolites in Five Woody Plant Species in the Boreal-Temperate Transition Zone
Source: Front Plant Sci. 2018 Sep 5;9:1257. doi: 10.3389/fpls.2018.01257 (PMC6134262; doi:10.3389/fpls.2018.01257)
Supplement: Supplementary file 3 [file Table_3.pdf]

Table S3. Results of linear mixed-effects models comparing changes in relative abundance of example compounds for different stress conditions. Statistically significant results ($\alpha = 0.05$) are identified with an asterisk (*) and change values preceded by "–" indicate a decline in mean relative abundance relative to our reference group, where as a "+" indicates an increase in mean relative abundance.

species	compound	year	stress condition	df	change (%)	t	P
balsam fir	resin acid 1	1	moderate temperature	29	-16.5	-0.724	0.4751
		1	high temperature	29	-15.9	-0.372	0.7126
		2	drought	25	+21.9	0.795	0.4343
		2	temperature	25	+28.7	1.238	0.2272
		2	drought + temperature	25	+30.0	1.234	0.2288
		3	light	8	+83.7	0.977	0.3570
		3	temperature	8	+120.2	1.484	0.1761
		3	temperature + light	8	+70.2	0.878	0.4057
	resin acid 2	1	moderate temperature	29	-14.2	-1.068	0.2944
		1	high temperature	29	-13.6	-0.919	0.3657
		2	drought	25	-3.0	-0.103	0.9188
		2	temperature	25	+5.4	0.333	0.7417
		2	drought + temperature	25	+13.1	0.736	0.4685
		3	light	8	-17.9	-0.586	0.5739
		3	temperature	8	+20.8	0.815	0.4389
		3	temperature + light	8	+39.5	1.495	0.1734
paper birch	catechin	1	moderate temperature	26	-54.3	-3.933	*< 0.0001**
		1	high temperature	26	-66.4	-4.322	*0.0002**
		2	drought	30	-23.9	-1.070	0.2931
		2	temperature	30	-33.8	-1.618	0.1161
		2	drought + temperature	30	-32.2	-1.489	0.1469
		3	light	6	+22.0	0.310	0.7670
		3	temperature	6	+44.5	0.626	0.5538
		3	temperature + light	6	+251.1	2.837	*0.0297**
	terpene acid	1	moderate temperature	26	-75.8	-3.015	*0.0057**
		1	high temperature	26	-71.4	-2.877	*0.0079**
		2	drought				
		2	temperature		*feature undetected*		
		2	drought + temperature				
		3	light	6	-98.0	-1.504	0.1832
		3	temperature	6	24.9	0.283	0.7870
		3	temperature + light	6	149.1	1.664	0.1470
