# Supplementary material for: Combinations of Abiotic Factors Differentially Alter Production of Plant Secondary Metabolites in Five Woody Plant Species in the Boreal-Temperate Transition Zone
Source: Front Plant Sci. 2018 Sep 5;9:1257. doi: 10.3389/fpls.2018.01257 (PMC6134262; doi:10.3389/fpls.2018.01257)
Supplement: Supplementary file 4 [file Image_1.pdf]

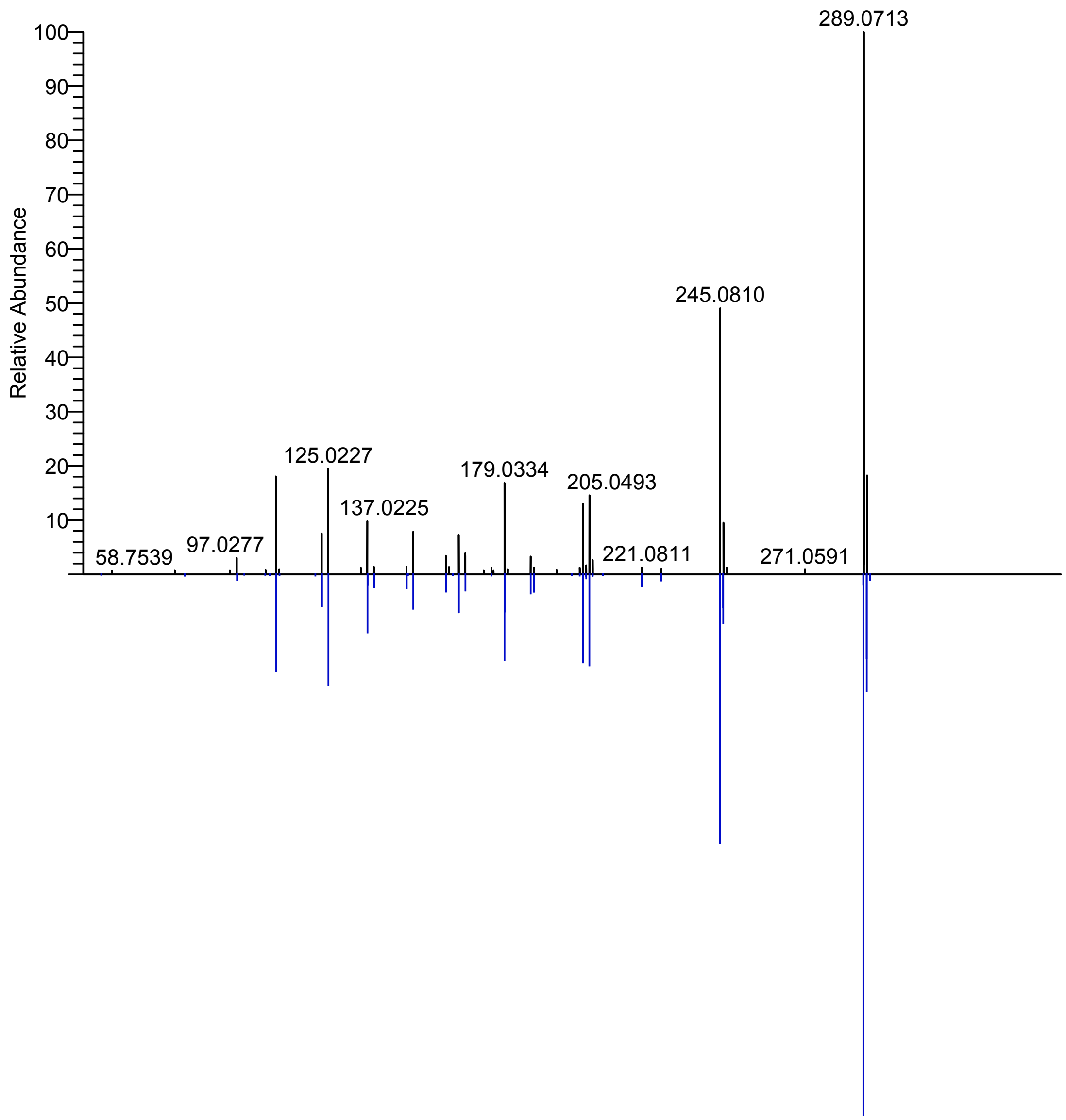

Figure S1. Mirrored HCD fragmentation spectra of endogenous catechin from Year 1 paper birch (above) and a catechin standard (below) from negative ionization mode. Catechin was identified and shown to be distinct from its isomer, epicatechin, as commercial standards of each of these compounds were chromatographically resolved. HCD fragmentation was performed at a normalized collision energy of 25.
