# Supplementary material for: Combinations of Abiotic Factors Differentially Alter Production of Plant Secondary Metabolites in Five Woody Plant Species in the Boreal-Temperate Transition Zone
Source: Front Plant Sci. 2018 Sep 5;9:1257. doi: 10.3389/fpls.2018.01257 (PMC6134262; doi:10.3389/fpls.2018.01257)
Supplement: Supplementary file 5 [file Image_2.pdf]

(A)

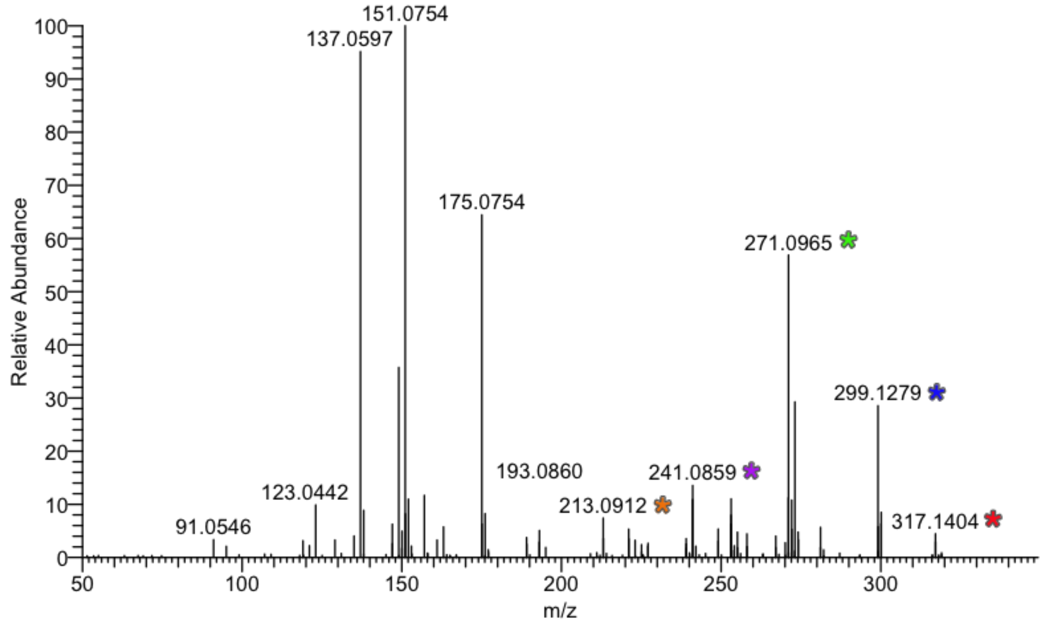

(B)

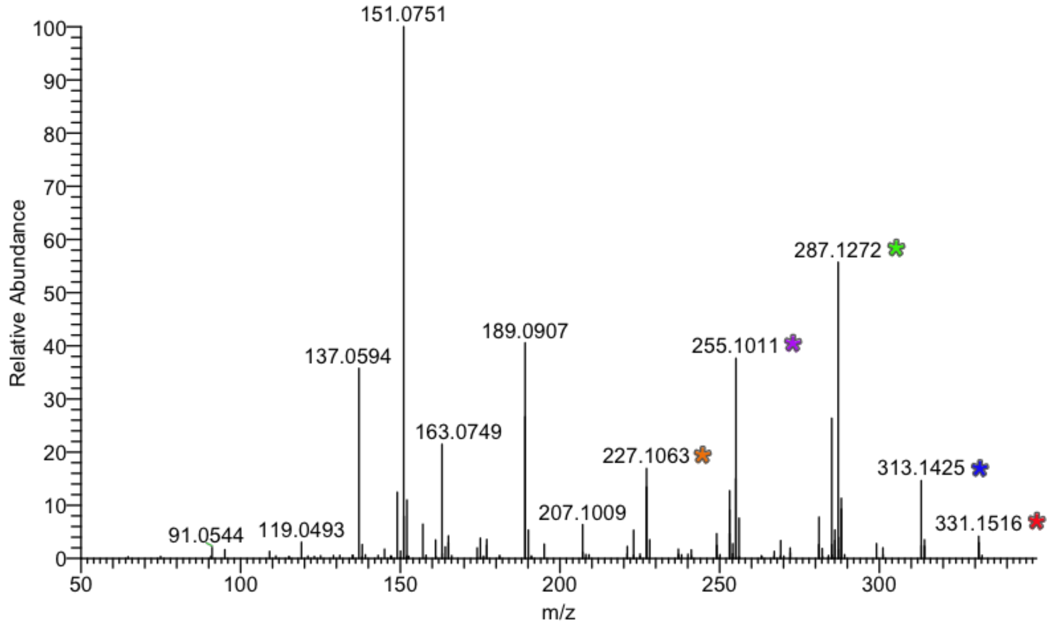

Figure S2. Positive ionization mode HCD fragmentation spectra of putative diterpene resin acids with *m/z* values of 317.1382 (A) and 331.1542 (B) from balsam fir. HCD fragmentation was performed at a normalized collision energy of 10. The spectra are very similar except for a 14 AMU shift (denoted with a *), suggesting these molecules are structurally related and differ only in the length of a hydrocarbon chain or presence/absence of a methylation.
