# Supplementary material for: Combinations of Abiotic Factors Differentially Alter Production of Plant Secondary Metabolites in Five Woody Plant Species in the Boreal-Temperate Transition Zone
Source: Front Plant Sci. 2018 Sep 5;9:1257. doi: 10.3389/fpls.2018.01257 (PMC6134262; doi:10.3389/fpls.2018.01257)
Supplement: Supplementary file 6 [file Image_3.pdf]

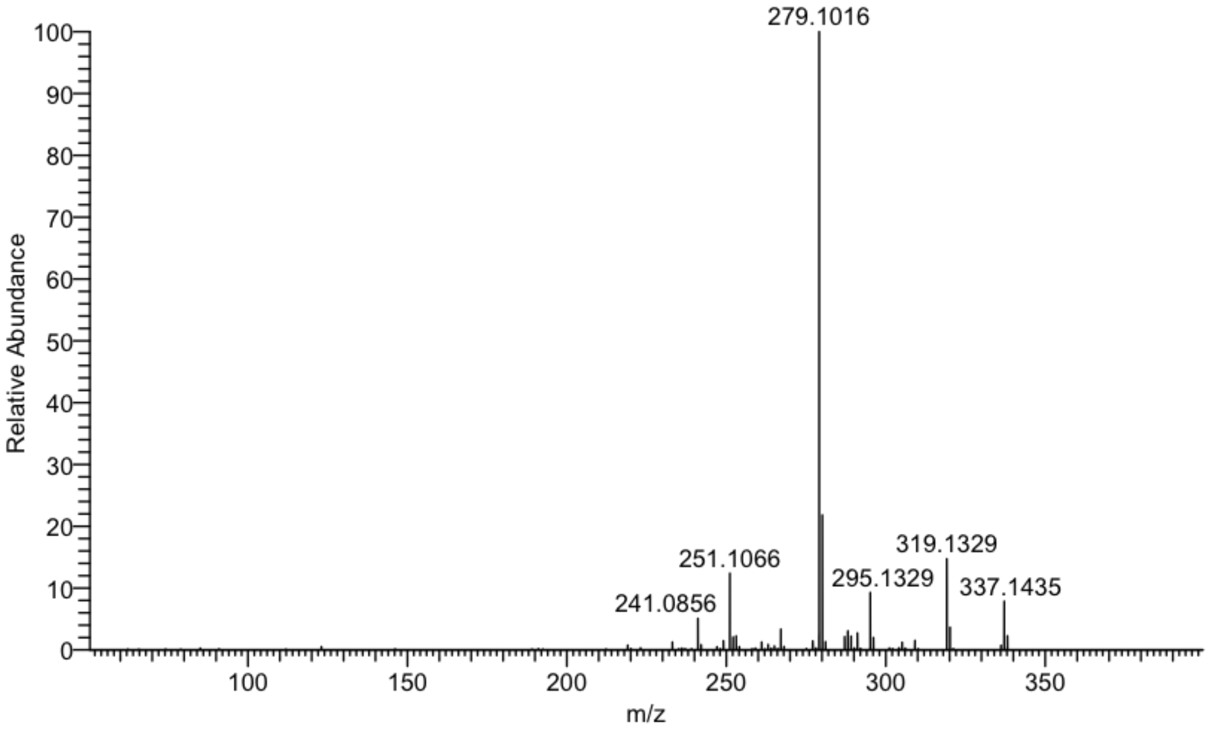

Figure S3. Positive ionization mode HCD fragmentation spectra of putative diterpene resin acid from paper birch. HCD fragmentation was performed at a normalized collision energy of 25.
