# Supplementary material for: Combinations of Abiotic Factors Differentially Alter Production of Plant Secondary Metabolites in Five Woody Plant Species in the Boreal-Temperate Transition Zone
Source: Front Plant Sci. 2018 Sep 5;9:1257. doi: 10.3389/fpls.2018.01257 (PMC6134262; doi:10.3389/fpls.2018.01257)
Supplement: Supplementary file 7 [file Image_4.pdf]

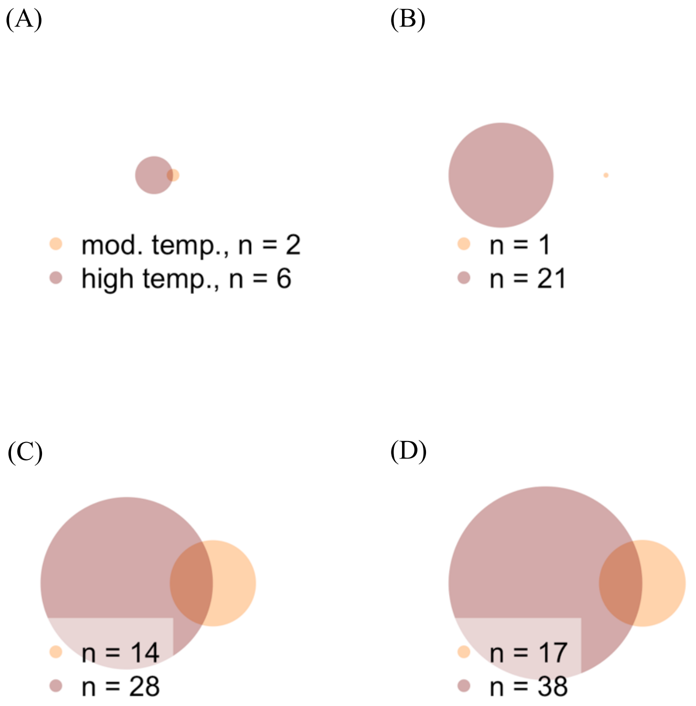

Figure S4. Venn diagrams for Year 1 samples detailing the number of compounds that increase or decrease by ≥ 75% in balsam fir (A and B, respectively) and paper birch (C and D, respectively). Circles are scaled and comparable across species and treatments. Areas in which circles are overlapping are relative to the number of compounds effected by both treatments. High-temperatures appears to have a greater influence on large scale shifts in the relative abundance of compounds than moderate temperatures.
