# Supplementary material for: Combinations of Abiotic Factors Differentially Alter Production of Plant Secondary Metabolites in Five Woody Plant Species in the Boreal-Temperate Transition Zone
Source: Front Plant Sci. 2018 Sep 5;9:1257. doi: 10.3389/fpls.2018.01257 (PMC6134262; doi:10.3389/fpls.2018.01257)
Supplement: Supplementary file 8 [file Image_5.pdf]

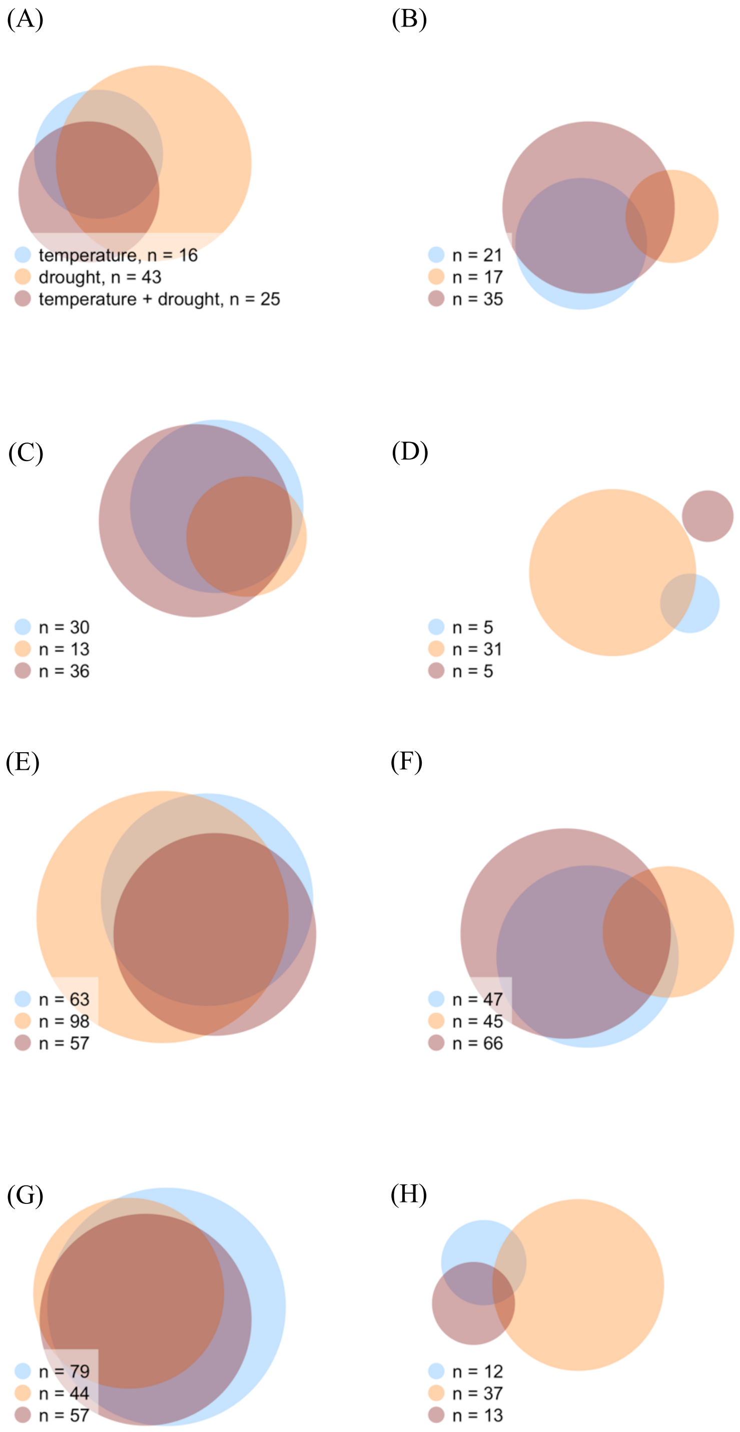

Figure S5. Venn diagrams for Year 2 samples detailing the number of compounds that increase or decrease by ≥ 75% in balsam fir (A and B, respectively), red maple (C and D, respectively), paper birch (E and F, respectively), and trembling aspen (G and H, respectively). Circles are scaled and comparable across species and treatments. Areas in which circles are overlapping are relative to the number of compounds affected by all treatments.
