# Supplementary material for: Combinations of Abiotic Factors Differentially Alter Production of Plant Secondary Metabolites in Five Woody Plant Species in the Boreal-Temperate Transition Zone
Source: Front Plant Sci. 2018 Sep 5;9:1257. doi: 10.3389/fpls.2018.01257 (PMC6134262; doi:10.3389/fpls.2018.01257)
Supplement: Supplementary file 9 [file Image_6.pdf]

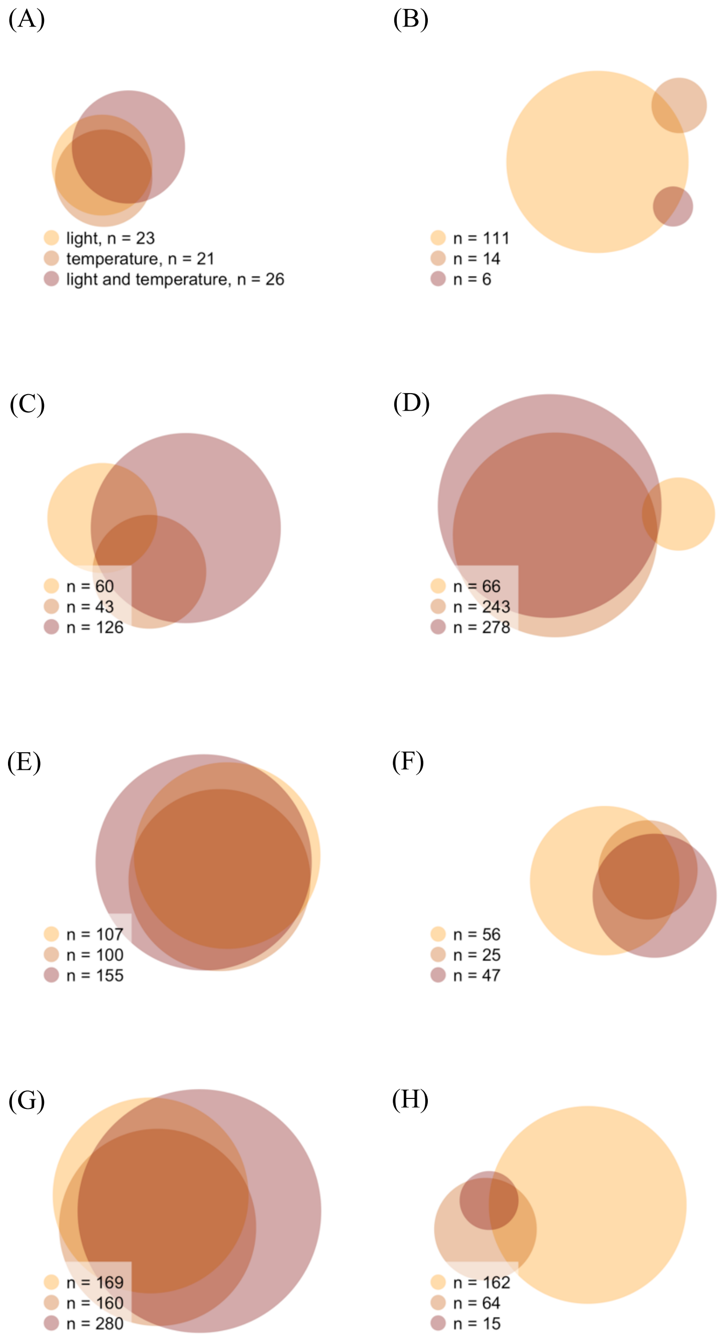

Figure S6. Venn diagrams for Year 3 samples detailing the number of compounds that increase or decrease by ≥ 75% in balsam fir (A and B, respectively), paper birch (C and D, respectively), beaked hazel (E and F, respectively), and trembling aspen (D and H, respectively). Circles are scaled and comparable across species and treatments. Areas in which circles are overlapping are relative to the number of compounds affected by all treatments. In general, the combination of high-light and high-temperature results in the large-scale increase of more compounds, on average, than any other treatment. While high-light conditions result in the large-scale decrease of more compounds, on average, than any other treatment.
