# Supplementary material for: Combinations of Abiotic Factors Differentially Alter Production of Plant Secondary Metabolites in Five Woody Plant Species in the Boreal-Temperate Transition Zone
Source: Front Plant Sci. 2018 Sep 5;9:1257. doi: 10.3389/fpls.2018.01257 (PMC6134262; doi:10.3389/fpls.2018.01257)
Supplement: Supplementary file 10 [file Image_7.pdf]

References

PRISM Climate Group (2017). *PRISM Climate Group*. Available at: http://prism.oregonstate.edu.

Sumner, L. W., Amberg, A., Barrett, D., Beale, M. H., Beger, R., Daykin, C. A., et al. (2007). Proposed minimum reporting standards for chemical analysis. *Metabolomics* 3, 211–221.
